# Development and psychometric evaluation of a questionnaire to measure university students’ knowledge on the effects of alcohol use during pregnancy

**DOI:** 10.3389/fpubh.2024.1399333

**Published:** 2024-05-10

**Authors:** Guilherme Petek Ramos Leite, Lucimar Retto da Silva de Avó, Carla Maria Ramos Germano, Débora Gusmão Melo

**Affiliations:** ^1^Departamento de Medicina, Universidade Federal de São Carlos (UFSCar), São Paulo, Brazil; ^2^National Institute on Population Medical Genetics (INAGEMP), Porto Alegre, Brazil; ^3^Departamento de Morfologia e Genética, Escola Paulista de Medicina, Universidade Federal de São Paulo (UNIFESP), São Paulo, Brazil

**Keywords:** alcohol consumption, pregnancy, fetal alcohol spectrum disorders, fetal alcohol syndrome, health knowledge, students, questionnaire design, Brazil

## Abstract

**Introduction:**

Alcohol consumption during pregnancy can lead to fetal alcohol spectrum disorders. This study developed and validated a questionnaire to assess university students’ knowledge regarding the effects of alcohol during pregnancy.

**Methods:**

We designed an instrument with true-false-I do not know statements. Initially, 45 true statements were formulated and subjected to content validation by 19 experts. Based on the Content Validity Index (CVI), 17 items were selected. The instrument, called the Fetal Alcohol Consequences Test (FACT), was first assessed by 31 university students for the level of understanding. Then, the questionnaire was administered to a national Brazilian sample of university students, and an Exploratory Factor Analysis (EFA) was conducted. Each correct FACT answer was worth 1 point, and the knowledge was categorized as high (total score ≥ 80%), moderate (score between 60 and 79%), and low (score ≤ 59%).

**Results:**

When the questionnaire was being designed, the CVI values ranged from 0.779 to 1.0, and all statements were considered suitable by the target audience. For psychometric evaluation, 768 students from 24 Brazilian states participated. In the EFA, five statements were removed, revealing a tool with 12 items and two latent factors: “fetal alcohol spectrum disorders” and “conceptions and guidance on alcohol consumption during pregnancy.” The KMO index (0.76426) and Bartlett’s sphericity test (6362.6, df = 66, *p* < 0.00001) both supported the final EFA model. The goodness-of-fit indices for the factor structure were adequate: *χ*^2^ = 119.609, df = 43, *p* < 0.00001; RMSEA = 0.048; CFI = 0.977; TLI = 0.965. The mean total FACT score among participants was 7.71 ± 2.98, with a median of 8; 32.03% of the students had high (10–12 points), 24.09% moderate (8–9 points), and 43.88% low knowledge (<8 points). The questionnaire proved reliable, with a floor effect of 1.17%, a ceiling effect of 9.25%, and a Cronbach’s alpha index of 0.798.

**Conclusion:**

The FACT can be utilized in university students’ health education processes, contributing to greater knowledge and information dissemination about the effects of alcohol during pregnancy, in addition to the formulation of policies on the subject directed to this group of young adults.

## 1 Introduction

Alcohol consumption during pregnancy can cause various types of embryo-fetal damage, which is why it is classified as a chemical teratogen (1). The mechanisms by which alcohol exerts its teratogenic role include epigenetic changes and disrupted development, brain injury, disruption of morphogens and growth factors, disruption of neuronal and glial migration, effects on neural stem cells, disruption of neuronal–glial interactions, neuroinflammation, gut microbiota alterations, and placental effects (2).

A distinct phenotype in children whose mothers consumed alcohol during pregnancy was defined in the 1970s and named “fetal alcohol syndrome” (FAS) (3). FAS is an irreversible condition characterized by craniofacial dysmorphia, intra-and extrauterine growth deficiencies, neurodevelopmental disorders, and various birth defects, most notably cardiac, renal, vertebral, and hearing disorders. The term “fetal alcohol spectrum disorders” (FASD) was coined later and is considered an umbrella term that encompasses all negative outcomes resulting from prenatal alcohol exposure (1, 2). Individuals with FASD can have a wide range of clinical phenotypes, ranging from FAS to congenital malformations and neurobehavioral disorders (4). The global prevalence of FASD is estimated at 19.0 per 1,000 individuals in the European region and 0.1 per 1,000 individuals in the Eastern Mediterranean region (2).

In line with the prevalence of FASD, the frequency of any amount of alcohol use during pregnancy among the general population is estimated at around 10%, varying from 25.2% in the European region to 0.2% in the Eastern Mediterranean region (2). In the Brazilian context, studies suggest that approximately 7–40% of women consume alcoholic beverages during pregnancy (5–11). This range has been attributed to the different instruments used to measure consumption and also to the period of pregnancy analyzed (5).

Greater consumption appears to be related to low education, inadequate housing conditions, low income, smoking, and the use of illicit drugs (5, 9–13). The literature discusses the risk associated with the marital situation, whereby studies show greater consumption among women who live without partners (5, 11, 13, 14) and others pointing to a greater risk among women with partners (9). There is also no consensus regarding the age risk. Although some studies indicate teenage pregnancy as being related to greater consumption (13), this association may not be due to maternal age but rather to the fact that the pregnancy was unplanned (12, 15).

The World Health Organization recommends total abstinence from alcohol consumption throughout pregnancy (16). In Brazil, the Ministry of Health reiterates that there is no safe amount of alcohol to consume during pregnancy and alerts the population of its deleterious fetal effects, therefore suggesting abstinence (17). Despite these recommendations, in practice, alcohol abuse during pregnancy is related to women’s individual reasons, as well as their knowledge and previous experiences with the subject. A permissive environment seems to favor consumption, emphasizing the importance of health education on the subject directed not only at women but also to the general population (18–20).

International studies, as well as a few studies conducted in Brazil, have investigated the general population’s knowledge and, particularly, women, whether pregnant or not, of the effects of alcohol consumption during pregnancy. The methodologies used in these studies vary widely, making comparisons difficult. In a study involving 221 postpartum women in South Korea, 86.9% of the participants reported that they had not received information on alcohol consumption during pregnancy and 12.7% continued drinking during their gestation (21). A Danish study conducted with 1,418 pregnant women showed that women under 25 years of age had a higher risk of not knowing health recommendations related to alcohol use during pregnancy (22). Among 1,237 pregnant women in Ethiopia, only 15.26% were informed about the risks of drinking alcohol by health care providers, and women who had lower knowledge levels on the harmful effects of alcohol consumption during pregnancy were 3.2 times more likely to drink alcohol compared to women who had a high level of knowledge (23). An Israeli study conducted with 802 pregnant women showed that the women who consumed alcohol in the 2 months before pregnancy knew less about the risks of such consumption than did the women who had not consumed alcohol (24). In Australia, a survey of 1,103 non-pregnant women showed that older women, with more children and less education, had less knowledge on the subject (25). In Russia, research carried out with 648 women showed that only 8% of women had accurate knowledge regarding fetal alcohol exposure (26). In Ghana, a study involving 294 women of reproductive age revealed that knowledge was directly proportional to the level of education, and participants who lived in rural areas had less knowledge (27). In general, research conducted with pregnant and postpartum women revealed that many participants did not receive guidance related to the subject during pregnancy from health professionals (20–24), and women with a lower level of education tend to have less knowledge on the theme (23, 25–27).

The topic is particularly relevant among students as young people are often heavy drinkers of alcohol and may have unprotected sex, leading to unplanned pregnancies (28). The understanding of high school pupils and university students on the topic has been explored in previous studies, indicating a general awareness regarding the harmful effects of alcohol during pregnancy (29–32). However, a smaller proportion of high school pupils and university students were familiar with the terms FASD, FAS, and their respective meanings (30–32). Among 1,035 American college students, 15% did not recognize the need for absolute abstinence throughout pregnancy (29). A Brazilian study with 331 university students enrolled in the first year of several health courses showed that 64.6% of participants were unaware of the harmful effects of alcohol on the fetus (30). In Italy, a survey carried out with 246 secondary school students noted that 30.1% of them believed that alcohol use was possible without damaging the fetus (31). Another Italian study with 9,921 secondary students showed that female and older students from Central and Northern Italy were better informed about gestational alcohol drinking risks (32).

In this context, we hypothesize that Brazilian university students have limited knowledge of the effects of alcohol consumption during pregnancy, which is a scientifically important public health issue. However, we did not identify valid instruments to investigate the subject in a systematic way. The purpose of this study was to develop and validate an instrument to measure university students’ knowledge about the effects of alcohol consumption during pregnancy that is user-friendly and can be answered quickly. A better understanding of students’ awareness of the subject can facilitate the development of more effective and culturally sensitive educational programs for this young population.

## 2 Materials and methods

### 2.1 Study design and ethical considerations

This is a descriptive and cross-sectional study that was conducted in two phases during August 2022 and January 2024. The questionnaire was developed in the first phase of the study, and the validity and reliability of the instrument were tested in the second phase using an exploratory factor analysis.

Ethical approval was obtained from the Human Research Ethics Committee at the Federal University of São Carlos (process CAAE 58094422.5.0000.5504) and all participants signed an informed consent. A data management plan for this research is available at https://doi.org/10.48321/D1QW4Z.

### 2.2 Phase 1: Development of the questionnaire

This research phase was conducted between August 2022 and March 2023. The questionnaire was developed in accordance with literature recommendations (33–38). The development phases followed the methodology proposed by Kishore et al. (38) and Azevedo and Scarpa (39). An advisory committee consisting of three of the authors (DGM, GPRL, and CMRG) was established. We chose to design an instrument with true-false-I do not know statements (40, 41) and a dichotomous score (each correct answer is equivalent to one point; wrong answers or “I do not know” do not score). The knowledge about the effects of alcohol use during pregnancy corresponds to the total score in the questionnaire.

Previously, a relevant literature review was conducted. For this purpose, the PubMed and SciELO databases were consulted. The literature review yielded information on the pathogenesis and clinical findings of FASD, the main beliefs and myths concerning alcohol consumption during pregnancy, the types of questions from existing questionnaires, and recommendations provided by various institutions regarding the topic. Thus, the researchers learned about the major themes in the different subject aspects.

Initially, 45 true statements were produced, and the content was validated by experts. The Content Validity Index (CVI), as proposed by Hernández-Nieto (42), was calculated for each item of the instrument. To do this, expert judges used a 5-point Likert scale to assess the level of language clarity and practical relevance of the 45 statements. The cutoff point adopted to determine satisfactory levels for language clarity and practical relevance was CVI ≥ 0.80. Additionally, the theoretical adequacy of each of the 45 questionnaire items was assessed using a dichotomous question of yes/no (42, 43). The experts also provided suggestions on how to better write the statements. These items were then analyzed and selected by the researchers. After this stage, 17 statements remained, six of which were transformed into false statements.

Following that, the instrument, entitled the Fetal Alcohol Consequences Test (FACT), was evaluated in relation to the level of understanding by the target audience. To achieve this, the questionnaire was administered to university students in the first and second years to check for difficulties and obtain suggestions on how to further clarify the statements. The students answered the following question: “Did you understand what was asked?” A 5-point Likert scale ranging from 0 to 4 was used; answers 3 and 4 were considered satisfactory, as suggested by Conti et al. (44, 45). The level of understanding of each item was calculated based on the arithmetic mean of the values given by the students. Changes to the wording of the statements were made based on student feedback. Additionally, the order of the 17 items was randomized following this stage to mitigate any potential bias among pretest respondents.

Since the targeted population for which the FACT was developed was Brazilian students, the original version of the instrument was in Portuguese. For the purpose of reporting, it was translated into English and reviewed by a native English language expert. Therefore, the English version of the instrument, which is also presented in this paper, was not culturally adapted.

### 2.3 Phase 2: Psychometric evaluation of the FACT questionnaire

A pretest was conducted to evaluate the psychometric properties of the semifinal version of the FACT. The invitation to participate in this study was sent to all Brazilian federal universities and was also heavily publicized on social networks (Facebook and Instagram). The inclusion criteria were Brazilian individuals, aged 18 years or older, who attended a university course at an institution in Brazil. The investigation was therefore carried out on a non-probability convenience sample (46).

The data were anonymously collected from April 2023 to August 2023 using a self-reported online form. In addition to the FACT, sociodemographic information was obtained using a form prepared for this study (Appendix S1), and the Brazilian version of the Sexual Transmitted Disease-Knowledge Questionnaire (STD-KQ) was applied (47–49), which was used for the FACT external construct validity assessment. The STD-KQ is a true-false-I do not know, comprehensive sexual transmitted infection knowledge questionnaire developed by American researchers in 2007 (47) and adapted and validated in Brazilian Portuguese (48, 49). The Brazilian questionnaire has 23 items; each correct item is worth one point, and the overall knowledge about sexually transmitted infections corresponds to the total score in the questionnaire.

To calculate the sample size, a proportion of at least 25 participants was considered for each item in the FACT, higher than the general recommendation of 10:1 found in the literature (50), which allows for more accurate exploratory factor analysis.

#### 2.3.1 Data analyses

The data analysis and the discussion of the results were conducted between August 2023 and January 2024. Descriptive analyses were performed for the characteristics of the pretest participants. Results were presented as the percentage, mean ± standard deviation (SD), and median (Mdn), depending on the variable.

An exploratory factor analysis (EFA) was conducted to evaluate the validity of the FACT internal construct. The FACTOR software (51), which we used to perform the EFA, offers several goodness-of-fit indices that are usually only seen in the confirmatory factor analysis (CFA). By supplementing the EFA with these indices, we reproduced a partial confirmatory factor analysis (PCFA). The primary utility of a PCFA is that, even when conducting a conventional EFA, we are able to obtain more convincing information as to whether considering a CFA of the model in the future is justifiable. Thus, these goodness-of-fit indices help to justify the recommendation of testing an EFA-derived model via CFA (52, 53). The validity and reliability of the FACT were measured in its original language, that is, in Portuguese.

The Kaiser–Meyer–Olkin index (KMO) > 0.5 and Bartlett’s sphericity test with a *p*-value <0.05 were considered prerequisites for determining whether the matrix was factorable (50). The number of retained factors was determined using the parallel analysis technique with a random permutation of the raw data (54). To complement the testing of the number of factors of the total instrument, unidimensionality/multidimensionality techniques were applied: Unidimensional Congruence (UniCo), Explained Common Variance (ECV) and Mean of Item REsidual Absolute Loadings (MIREAL) (55). The EFA was performed using a polychoric matrix and the Robust Diagonally Weighted Least Squares (RDWLS) as the method for factor extraction (56). As a rotation technique, we used the Robust Promin (57).

The adequacy of the model was evaluated using the following goodness-of-fit indices: Root Mean Square Error of Approximation (RMSEA <0.08), Comparative Fit Index (CFI > 0.90), and Tucker–Lewis Index (TLI > 0.90) (58). The psychometric robustness of the model was assessed through bootstrap validation (500 resamples) that was used to generate a confidence interval (CI) for goodness-of-fit indices. Searching for the best factorial model, the following criteria were used to remove items: low Measure of Sampling Adequacy (MSA) value (<0.25), low factor loading (<0.3), presence of cross-loading (difference between factor loadings <0.15), and low communality (<0.25) (59–61).

Regarding the quality and effectiveness of factor score estimates, accuracy (Overall Reliability of fully Informative prior Oblique N-EAP scores – ORION >0.80), representativeness of the latent trait and effectiveness of factor estimation (Factor Determinacy Index – FDI > 0.90), sensitivity (Sensitivity Ratio – SR > 2.0), and the expected percentage of the factor (Expected Percentage of true Differences – EPTD >90%) were assessed. Composite reliability, calculated by the Composite Reliability Calculator, was based on standardized factor loadings and error variances (62, 63); the reference values adopted for these measures were <0.6 low, between 0.6 and 0.7 moderate, and between 0.7 and 0.9 high reliability (64). The stability of the factors was evaluated using Generalized H indices; values of G-H > 0.80 suggest a well-defined latent variable, which is more likely to be stable in different studies, that is, replicable (55).

After carrying out the EFA and considering the results of the FACT best model, the questionnaire’s reliability was evaluated in terms of internal consistency using Cronbach’s alpha index, and values ≥0.70 were considered adequate (65). Descriptive analyses of FACT results were carried out. A response frequency diagram was constructed for each question, and the percentage of correct answers for each item and the general questionnaire was calculated. Floor and ceiling effects were evaluated by calculating the percentages of the responses with the lowest or highest possible scores; rates greater than 15% for the highest and lowest scores indicated ceiling and floor effects, respectively (66). Knowledge about the effects of alcohol use during pregnancy was categorized into three levels using the original Bloom’s cut-off points: high knowledge if the total score was between 80 and 100%; moderate knowledge if the total score was between 60 and 79%; and low knowledge if the total score was ≤59% (67).

The normality of the FACT total score was verified using the Kolmogorov–Smirnov test with Lilliefors correction. Since the normality of the FACT total score was rejected (*D* = 0.1119; *p* < 0.0001), non-parametric statistical methods were used. The convergent validity of the FACT was determined through the correlation with the STD-KQ using the Spearman correlation coefficient (rho), interpreted as: 0.00 to 0.10—negligible correlation; 0.10 to 0.39—weak correlation; 0.40 to 0.69—moderate correlation; 0.70 to 0.89—strong correlation; and 0.90 to 1.0—very strong correlation analysis (68). The difference of the FACT scores according to the sociodemographic characteristics of the participants was analyzed using the Mann–Whitney or Kruskal–Wallis tests with Dunn’s post-test, depending on the number of groups in each variable.

Statistical analyses were performed using JASP 0.18.1 (69), MedCalc 22.014 (70) and FACTOR 12.04.01 (51). A *p*-value of <0.05 was considered statistically significant.

## 3 Results

### 3.1 Development of the FACT questionnaire

After a bibliographical review, the 45 true statements were prepared by the authors (Supplementary Table S1) with approximately the same size, seeking to avoid the tendency of respondents to consider a larger text as correct. The language was adapted to the target audience. All statements addressed topics related to the research themes: three epistemological topics and one topic related to myths and misconceptions, presented, respectively, in Supplementary Tables S2, S3.

Content validation was performed on these statements by 10 geneticists, 3 pediatricians, 3 obstetrician-gynecologists, and both 3 geneticists and pediatricians. Regarding language clarity, the statements achieved CVI values ranging between 0.779 and 0.979. Regarding practical relevance, CVI values varied between 0.842 and 1.0. The CVI results are detailed in Supplementary Table S4. In terms of theoretical adequacy, 13 of the 45 statements achieved adequacy lower than 90% (items 5, 6, 10, 12, 13, 21, 23, 26, 27, 28, 29, 35, and 43). Additionally, the judges provided some suggestions about the text writing of the statements.

During this process, the statements were ranked based on the content validation indexes (Supplementary Tables S5–S7), and those with low CVI values were changed or removed from the questionnaire. The statements that received negative criticism from the expert judges and those that, after a new evaluation by the advisory committee, were considered to be of low relevance or similar in meaning to other items already present in the instrument were also removed. The instrument was then reduced by the research advisory committee to 17 statements, with 6 statements transformed into false statements. Supplementary Table S8 presents FACT after expert content validation.

This instrument with 17 items was subjected to an evaluation of the level of understanding, in which 31 university students participated. At this stage, the assertions reached a level of understanding ranging between 3.77 and 4, while FACT as a whole achieved a level of understanding of 3.92 (Supplementary Table S9). The students made some suggestions about the writing of the statements, which were considered by the advisory committee. A semi-final version of the FACT was developed (Supplementary Table S10), consisting of 17 closed-ended items.

### 3.2 Psychometric evaluation of the FACT questionnaire

#### 3.2.1 Pretest participants

Initially, 785 students had joined the research. From the initial pool, 5 were removed as they were foreign students, 10 because they were under 18, and 2 due to providing incomplete responses. In total, 768 undergraduate students participated in the research, of whom 72.14% (*n* = 554) were female and 27.86% (*n* = 214) were male. These participants came from 24 states, with a significant predominance in São Paulo, representing 64.6% (*n* = 496). The mean age of the respondents was 24.03 ± 6.62 years. Regarding skin color/ethnicity, the majority (67.06%, *n* = 515) identified as white, followed by 21.62% (*n* = 166) as mixed-race, 6.51% (*n* = 50) as black, 4.04% (*n* = 31) as Asian, and 0.78% (*n* = 6) as indigenous.

Concerning marital status, the majority of students (78.39%, *n* = 602) declared themselves as single. With respect to sexual orientation, 66.41% (*n* = 510) identified as heterosexual, 6.64% (*n* = 51) as homosexual, 19.66% (*n* = 151) as bisexual, 2.34% (*n* = 18) as pansexual, 2.47% (*n* = 19) as asexual, and 2.47% (*n* = 19) did not want to share information. The majority, 88.80% (*n* = 682), did not have children. Among the female participants, 87.36% (*n* = 484) had never been pregnant, 8.12% (*n* = 45) had one pregnancy, 3.07% (*n* = 17) had two pregnancies, and 1.44% (*n* = 8) had three or more pregnancies. Regarding religion, 45.44% (*n* = 349) declared themselves as non-religious, while 28.78% (*n* = 221) identified themselves as Catholics.

The majority of respondents (90.23%; *n* = 693) attended public universities. As for the field of study, 28.78% (*n* = 221) were in math and science careers, 20.96% (*n* = 161) in humanities, 17.32% (*n* = 133) in biological sciences, and 32.94% (*n* = 253) in health sciences. Regarding the type of high school attended, 44.92% (*n* = 345) went to public school, 51.04% (*n* = 392) attended private school, and 4.04% (*n* = 31) attended both public and private schools. In terms of monthly income, most college students (37.89%, *n* = 291) earned 1–3 minimum wages (MW), 28.52% (*n* = 219) earned 4–6 MW, and 15.63% (*n* = 120) earned 7–10 MW. Supplementary Table S11 presents the sociodemographic information of the participants.

#### 3.2.2 Exploratory factor analysis

The sample size was appropriate for the EFA execution as it allowed 45 respondents per FACT item. Firstly, an EFA was conducted with all 17 FACT items. The KMO index was unacceptable (0.35755) and items 1 and 14 presented normed MSA values below 0.25 (0.15740 and 0.22011, respectively). In a second EFA model, after removing these two items with lower MSA values, the KMO value improved (0.80527). However, this new model showed item 5 with cross-loading and item 17 with factor loading below 0.30. Both items were removed and a new EFA was performed. In this third EFA model, item 13 displayed low communality (0.198) and also needed to be removed. Lastly, the final EFA model was carried out with 12 items, excluding items 1, 5, 13, 14, and 17, and was considered appropriate. Table 1 presents the FACT’s final version with 12 items.

**Table 1:** FACT’s final version, after exploratory factor analysis.

No.	Statements in Portuguese	Statements in English
1	Tomar bebida alcoólica durante a gravidez pode causar defeitos no coração da criança. (V)	Consuming alcoholic beverages during pregnancy can cause heart defects in the child. (T)
2	Tomar bebida alcoólica durante a gravidez pode causar problemas permanentes na criança, incluindo defeitos físicos e deficiência intelectual, que caracterizam a síndrome alcoólica fetal. (V)	Consuming alcohol during pregnancy can cause permanent problems in the child, including physical disorders and intellectual disability, which characterize fetal alcohol syndrome. (T)
3	Mães que beberam álcool na gravidez podem ter crianças com inteligência abaixo do normal. (V)	Mothers who had alcohol during pregnancy may have children with below-average intelligence. (T)
4	Mães que beberam álcool na gravidez podem ter crianças com problemas emocionais e de comportamento, como ataques de raiva ou choro, ansiedade e agressividade. (V)	Mothers who had alcohol during pregnancy may have children with emotional and behavioral problems, such as tantrums, crying, anxiety, and aggressiveness. (T)
5	Se uma mulher bebeu álcool durante a gravidez e seu filho nasceu saudável, isso indica que ela pode beber nas próximas gestações sem qualquer risco para a criança. (F)	If a woman had alcohol during her pregnancy, and her child was born healthy, it indicates that she can continue drinking it in subsequent pregnancies, as there are no risks for the child. (F)
6	Qualquer quantidade de álcool consumido pela gestante pode prejudicar o bebê, portanto, não há dose segura. (V)	Any amount of alcohol consumed by a pregnant woman can harm the baby, so there is no safe level of alcohol consumption. (T)
7	Qualquer tipo de bebida alcoólica consumida durante a gravidez pode prejudicar o desenvolvimento do bebê. (V)	Any type of alcohol consumed during pregnancy can be harmful to the baby’s development. (T)
8	Mães que beberam álcool na gravidez podem ter crianças com problemas no desenvolvimento neurológico, como falta de atenção e hiperatividade. (V)	Mothers who had alcohol during pregnancy may have children with neurological development disorders, such as attention deficit and hyperactivity. (T)
9	A cerveja preta melhora a quantidade do leite materno. (F)	Dark beer improves breast milk production. (F)
10	Os estudos científicos recomendam não consumir qualquer quantidade de álcool durante todo o período da gravidez. (V)	Scientific studies recommend not consuming any amount of alcohol throughout the entire pregnancy. (T)
11	O consumo de bebidas alcoólicas por mulheres grávidas pode prejudicar o desenvolvimento do bebê apenas nos três primeiros meses de gravidez. (F)	Pregnant women’s consumption of alcoholic beverages can harm the baby’s development only in the first three months of pregnancy. (F)
12	Tomar bebida alcoólica durante a gravidez pode causar diminuição da cabeça na criança (microcefalia). (V)	Consuming alcoholic beverages during pregnancy can cause a reduction in the size of the baby’s head (microcephaly). (T)

The letter “F” stands for false statements, whereas the letter “T” stands for true statements.

The KMO index (0.76426) and Bartlett’s sphericity test (6362.6, df = 66, *p* < 0.00001) both supported this final EFA model. The parallel analysis identified that two factors represented the data because two factors of the real-data presented a percentage of explained variance higher than the variance mean of the random data (Table 2). The total explained that the variance of these two FACT factors was 67.35%. The values of UniCo (0.936, <0.95), ECV (0.751, <0.85), and MIREAL (0.365, >0.300) confirmed that the pretesting data did not allow the FACT to be considered unidimensional.

**Table 2:** Results of the parallel analysis.

Factors	% of variance of real-data	% of variance of random data (mean)
1	**50.5198**	**17.0064**
2	**16.8261**	**14.9271**
3	8.4261	13.3106
4	6.8576	11.8574
5	4.7568	10.3876
6	3.9747	8.9756
7	3.5903	7.5804
8	2.5702	6.2155
9	1.9082	4.7189
10	0.3609	3.2873
11	0.2093	1.7331

Marked in bold are the percentages of variance of the two factors in the FACT questionnaire.

Each factor comprised six items: the first factor contained items 1, 2, 3, 4, 8, and 12, and was named “fetal alcohol spectrum disorders” while the second factor contained items 5, 6, 7, 9, 10, and 11, and was named “conceptions and guidance on alcohol consumption during pregnancy.” The 12 items of the FACT presented adequate factor loadings in their respective factors (Table 3).

**Table 3:** Factor loadings and communalities for the 12 items of the FACT, as well as quality and effectiveness of factor score estimates, composite reliability indices, and estimates of replicability of the two factors.

Items	Factor 1	Factor 2	*h*^2^
Item 1 – Alcohol and heart defects	0.081	**0.598**	0.872
Item 2 – Fetal Alcohol Syndrome	0.175	**0.666**	1.000
Item 3 – Alcohol and lower intelligence	0.081	**0.688**	0.890
Item 4 – Alcohol and behavioral problems	−0.192	**0.869**	0.765
Item 5 – Misconception about alcohol and past pregnancies	**0.409**	0.205	0.476
Item 6 – Any amount of alcohol is dangerous	**0.858**	−0.088	0.932
Item 7 – Any type of beverage is dangerous	**0.886**	0.009	1.000
Item 8 – Alcohol and neurological problems	−0.098	**0.948**	0.920
Item 9 – Misconception about dark beer and breast-feeding	**0.644**	0.069	0.608
Item 10 – Scientific studies and alcohol intake	**0.916**	−0.114	0.838
Item 11 – Alcohol in the first trimester	**0.458**	0.278	0.785
Item 12 – Alcohol and microcephaly	0.139	**0.623**	0.710
ORION	0.918	0.916	–
Factor Determinacy Index – FDI	0.958	0.957	–
Sensitivity ratio – SR	3.352	3.305	–
Expected percentage of true differences – EPTD	93.4%	93.3%	–
Composite reliability	0.859	0.878	–
GH-latent index	0.918	0.916	–
GH-observed index	0.681	0.749	–

*h*^2^, communality. Marked in bold are the six items of each factor in the FACT questionnaire.

Quality and effectiveness of factor score estimates, composite reliability indices, and estimates of the replicability of factorial scores (G-H indices) were also provided in Table 3. Both the generated factor scores were considered reliable because ORION values were above 0.80, FDI values were above 0.90, SR values were above 2, and EPTD indices were above 90%. The composite reliability of both factors was high (Factor 1 = 0.859 and Factor 2 = 0.878). As expected, the GH-latent values are higher than GH-observed values for both factors, reflecting the result that the factors are better defined by the underlying responses than by the observed item scores (55). Finally, the goodness-of-fit indices for the factor structure were adequate: *χ*^2^ = 119.609, df = 43, *p* < 0.00001; RMSEA = 0.048 (95% CI 0.0340–0.0535); CFI = 0.977 (95% CI 0.972–0.987); TLI = 0.965 (95% CI 0.956–0.980).

#### 3.2.3 Descriptive results of the FACT, ceiling and floor effects, and reliability statistics

Cronbach’s alpha index for the total FACT was 0.7976, with values ranging from 0.7473 to 0.7827 for each questionnaire item (Supplementary Table S12).

**Figure 1:**
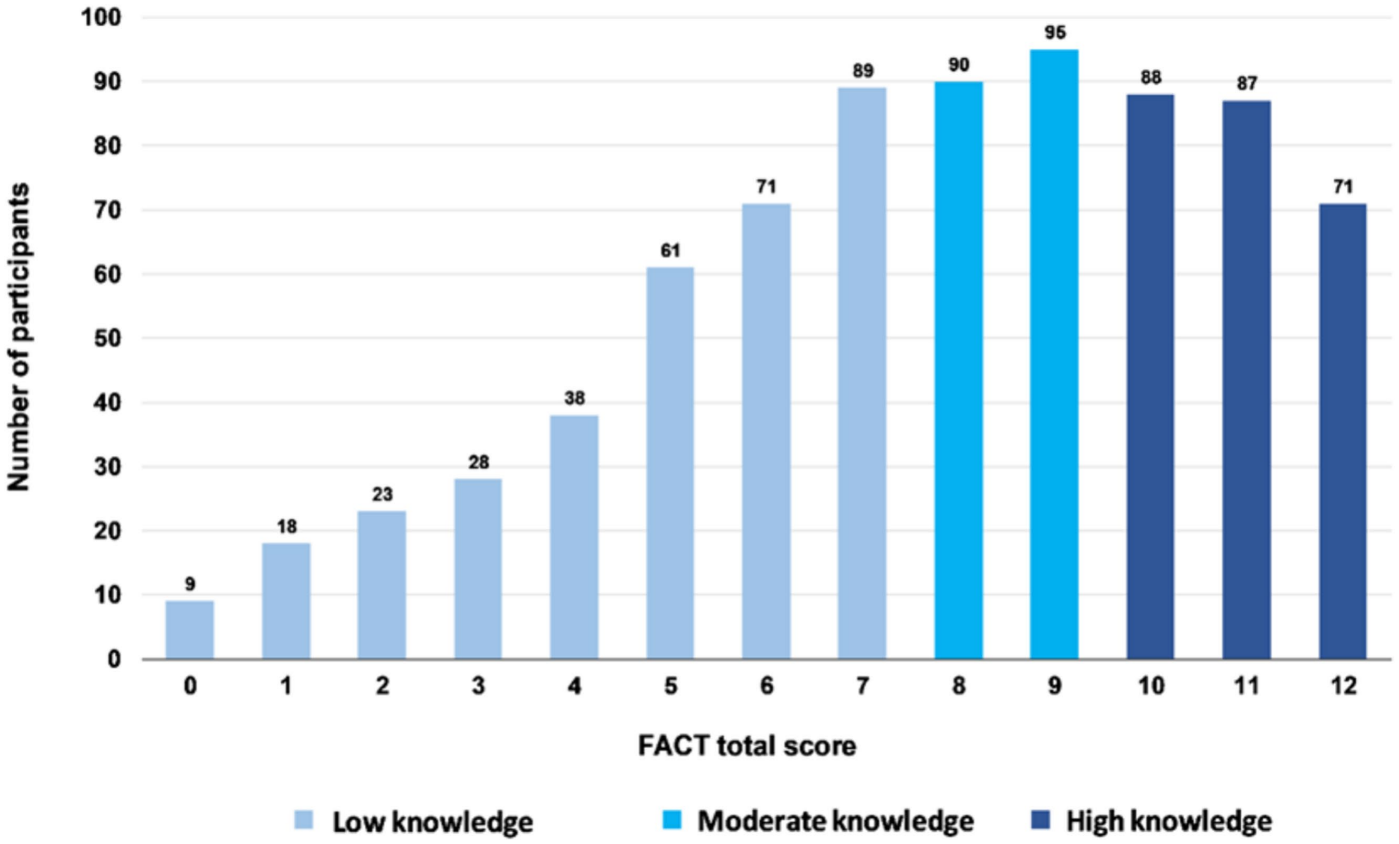
The FACT total score distribution in pretest participants (*n* = 768).

**Figure 2:**
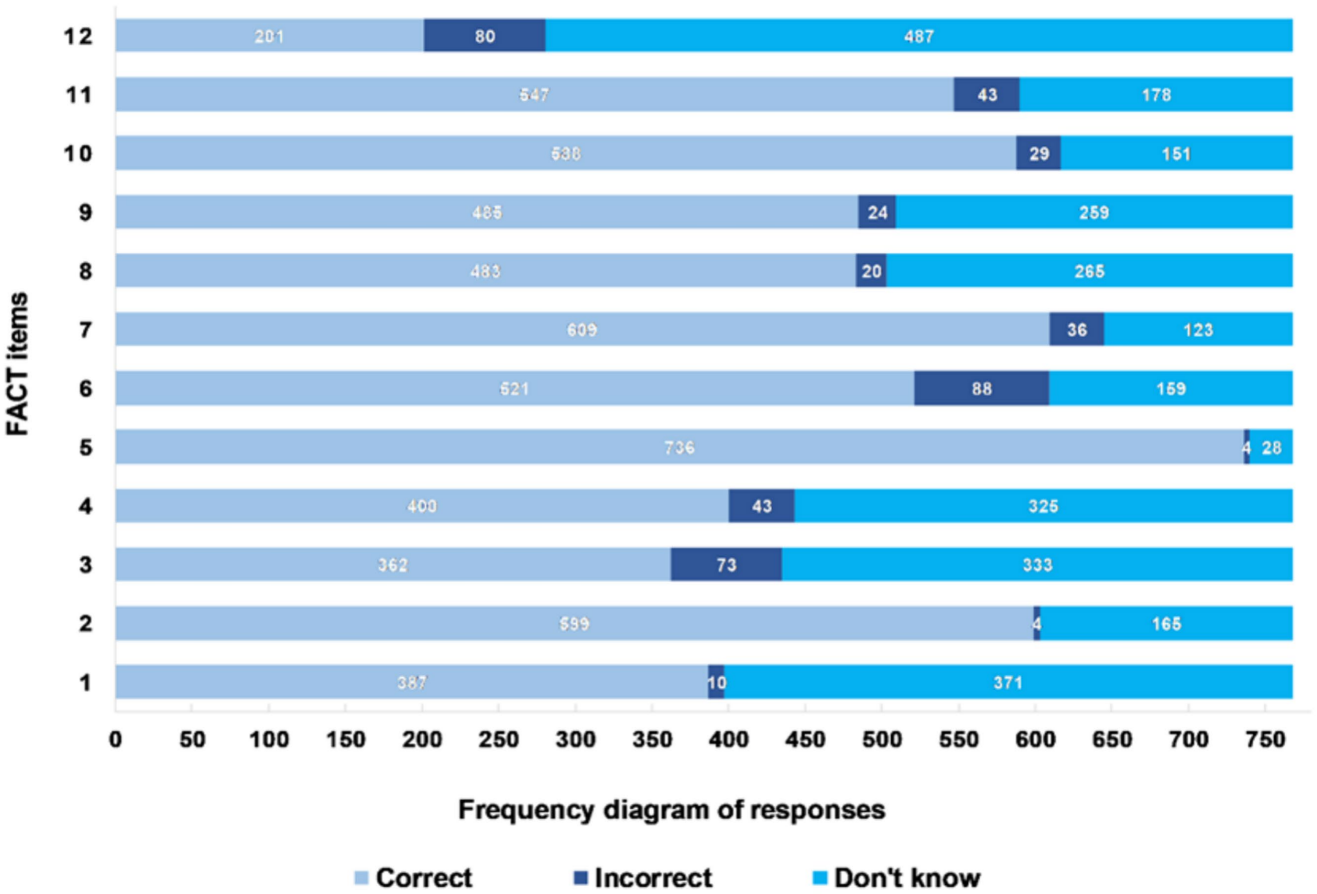
Frequency diagram of responses to the 12 items of the FACT among pretest participants (*n* = 768).

The mean total FACT score was 7.71 (± 2.98, 95% CI 7.50–7.92), with a median of 8, a minimum of 0, and a maximum of 12 points. The floor effect was 1.17% and the ceiling effect was 9.25%. Figure 1 depicts the results of the FACT total score among the pretest participants, while Figure 2 presents the frequency diagram of responses to the 12 items of the FACT.

Statements 5 (misconception about alcohol and past pregnancies), 7 (any type of beverage is dangerous), 2 (fetal alcohol syndrome), 10 (scientific studies and alcohol intake), and 11 (alcohol in the first trimester) exhibited, in descending order, the highest percentage of correct responses. They achieved success rates of 95.83, 79.30, 78.00, 76.56, and 71.22%, respectively. On the other hand, statements 8 (alcohol and neurological problems), 4 (alcohol and behavioral problems), 1 (alcohol and heart defects), 3 (alcohol and lower intelligence), and 12 (alcohol and microcephaly) showed, in descending order, the lowest accuracy rates, with success percentages of 62.89, 52.08, 50.39, 47.14, and 26.17%, respectively.

Using the three-level categorization as proposed by Bloom (67), 32.03% (*n* = 246) of the students had a high knowledge (10 to 12 points), 24.09% (*n* = 185) had a moderate knowledge (8–9 points), and 43.88% (*n* = 337) had a low knowledge (<8 points).

#### 3.2.4 External construct validity with the STD-KQ

Cronbach’s alpha index for the total STD-KQ was 0.8653, with values ranging from 0.8372 to 0.8489 for each questionnaire item (Supplementary Table S13). The mean total STD-KQ score was 15.14 (±5.29, 95% CI 14.76–15.51), with a median of 16, a minimum of 0, and a maximum of 23 points. The floor effect was 1.17% and the ceiling effect was 3.0%.

The FACT scores were positively and moderately correlated with the STD-KQ scores (rho = 0.427, *p* < 0.001), indicating that the more knowledge about alcohol consumption during pregnancy the students had, the more knowledge about sexually transmitted infections they also had.

### 3.3 Comparison of FACT scores among sociodemographic groups

Comparison of FACT scores among sociodemographic groups can be seen in Table 4. A Mann–Whitney test indicated that the FACT score was greater for female participants (Mdn = 8) than for male participants (Mdn = 7) (*U* = 66,703; *p* = 0.007).

**Table 4:** The FACT score distribution according to the sociodemographic (*n* = 768).

Variables	*n*	FACT score			*p-*value^†^
		Mean	*SD*	Median	
**Gender**					
Female	554	7.92	2.82	8	0.007^**^
Male	214	7.15	3.31	7	
**Age (years)**					
18–30	675	7.64	2.96	8	0.007^**^
>31–40	67	8.34	3.00	9	
>41–50	17	9.06	2.84	10	
>50	9	5.44	3.47	5	
**Skin color/ethnicity**					
White	515	7.79	3.01	8	0.597
Black	50	7.54	3.15	8	
Mixed-race	166	7.48	2.87	8	
Asian	31	7.58	2.74	8	
Indigenous	6	8.50	3.51	8.50	
**Sexual orientation**					
Heterosexual	510	7.72	3.03	8	0.883
Homosexual	51	7.84	3.01	8	
Bisexual	151	7.72	2.82	8	
Pansexual	18	7.06	3.17	6.50	
Asexual	19	7.11	3.30	8	
Do not want to share	19	8.00	2.36	8	
**Marital status**					
Single	602	7.61	2.97	8	0.003^**^
Married	72	8.57	3.21	10	
Common-law marriage	86	7.90	2.67	8	
Divorced	8	5.50	3.30	5	
**Previous pregnancy (females only)**					
0	695	7.53	3.01	8	0.001^**^
1	47	9.28	1.90	10	
2	18	9.33	2.61	10	
3 or more	8	10.00	2.20	11	
**Alive children**					
0	682	7.59	2.97	8	0.003^**^
1	58	8.74	2.61	9.50	
2	21	7.81	3.43	9	
3 or more	7	10.00	3.16	11	
**Type of high school**					
Public	345	7.47	3.07	8	0.030^*^
Private	392	7.97	2.89	8	
Public and private	31	6.97	2.82	7	
**Type of university/college**
Public	693	7.68	2.99	8	0.533
Private	75	7.91	2.93	8	
**Field of study**
Health Sciences	253	8.63	2.71	9	0.001^**^
Math and Science	221	6.95	3.09	7	
Humanities	161	7.32	3.15	8	
Biological Sciences	133	7.68	2.61	8	
**Monthly income (MW = minimum wage)**
1–3 MW	291	7.52	3.01	8	0.265
4–6 MW	219	7.66	2.92	8	
7–10 MW	120	7.84	2.98	8	
11–13 MW	54	7.69	2.77	8	
14–16 MW	23	8.09	3.26	9	
Over 16 MW	61	8.39	3.13	9	
**Religion**					
Catholic	221	7.85	2.95	8	0.204
Evangelical	108	7.83	3.00	8	
African-based religion	25	8.84	2.01	9	
Non-religious	349	7.49	3.01	8	
Others	65	7.74	3.15	8	
**Region of the country**					
Southeast	520	7.71	2.94	8	0.408
South	45	7.87	2.97	8	
Midwest	62	7.34	3.33	8	
Northeast	102	7.56	2.97	8	
North	39	8.49	3.01	9	

^†^Mann–Whitney or Kruskal–Wallis tests; ^*^p < 0.05, ^**^p < 0.01.

There was a statistically significant difference between the FACT score by age [*H*(3) = 12.240; *p* = 0.007], marital status [*H*(3) = 13.618; *p* = 0.003], previous pregnancy [*H*(3) = 27.543; *p* < 0.001], alive children [*H*(3) = 14.003; *p* = 0.003], type of high school [*H*(2) = 7.012; *p* = 0.030], and field of study [*H*(3) = 40.971; *p* < 0.001].

Concerning age, the post-test showed a difference between the “18 to 30 years” group compared to the “31 to 40 years” (*p* = 0.048), “41 to 50 years” (*p* = 0.039), and “above 50 years” (*p* = 0.045) groups. There was also a difference between the “31 to 40 years” and “41 to 50 years” groups in relation to the “above 50 years” group (*p* = 0.009 and *p* = 0.004, respectively). In terms of marital status, there was a difference between the “single” and “married” groups (*p* = 0.002), the “married” and “divorced” groups (*p* = 0.004), and the “common-law marriage” and “divorced” groups (*p* = 0.039). Regarding previous pregnancy, differences were observed between the group of people who have never been pregnant compared to the groups who have been pregnant once (*p* < 0.001), twice (*p* = 0.006), or three or more times (*p* = 0.012). Concerning the number of alive children, differences were noted between the group of those who do not have alive children compared to those who have one alive child (*p* = 0.004) and those who have three or more alive children (*p* = 0.015). Regarding the type of high school, differences were found between the “public” and “private” groups (*p* = 0.035) and between the “private” and “both, public and private” groups (*p* = 0.05). In terms of the field of study, differences existed between the “health sciences” and “math and science” groups (*p* < 0.001), “health sciences” and “humanities” groups (*p* < 0.001), and “health sciences” and “biological sciences” groups (*p* = 0.004).

## 4 Discussion

This study developed and validated an instrument to assess university students’ knowledge about the effects of alcohol during pregnancy. In this regard, the FACT is the first tool created in Brazil specifically for this purpose.

During the process of creating the first 45 statements, we reviewed articles on the subject from America, Africa, Europe, Oceania, and Asia, and we noted that several issues were addressed similarly across each study. These issues were categorized into epistemological topics and myths and misconceptions that permeate the use of alcohol during pregnancy. Subsequently, we adapted this categorization to the Brazilian sociocultural context and used it to construct the statements.

Three of the 45 statements (items 16, 33, and 43) achieved language clarity levels below acceptable in the FACT content validation process. We believe that the expressions “placental barrier” (statement 16) and “genetic profile” (statement 33) have been considered hermetic and, therefore, of low clarity. In statement 43, the double negation in “it has none” (in Portuguese: “não tem nenhuma”), although often used in colloquial Brazilian Portuguese, may have affected its clarity. All assertions reached the minimum desired CVI value in relation to practical relevance, which demonstrates the importance of all epistemological themes and myths/misconceptions listed in the research. The expert judges considered 13 of the 45 statements as covering knowledge not appropriate to be evaluated by the target audience, therefore achieving levels of theoretical adequacy below what is desirable. The results of the CVI and the suggestions received by the expert judges supported the changes to the FACT statements made by the advisory committee. As a consequence of that, in the stage of evaluating the level of understanding of FACT among the target population, all 17 items were considered adequate.

Although four distinct theoretical issues (three epistemological topics and one topic related to myths and misconceptions) guided the construction of the 45 initial FACT statements, the EFA indicated a factorial solution with two latent factors (“fetal alcohol spectrum disorders” and “conceptions and guidance on alcohol consumption during pregnancy”). The topic “fetal alcohol spectrum disorders” emerged as a factor, while the other topics converged in a second factor entitled “conceptions and guidance on alcohol consumption during pregnancy.” This factorial solution presented excellent explained variance (71), which indicates that the proposed model elucidated a significant part of the variance in the data set.

In psychometric terms, the factorial solution found was considered robust, with an adequate sample size and with extraction, retention, and factor rotation methods recommended by current literature (72). Regarding reliability, the FACT presented adequate internal consistency both in the general instrument and in each of the statements separately (65). The other goodness-of-fit indices were also within reference values, which strengthens the factorial model developed.

In spite of the factorial model’s good adequacy, it is noteworthy that future studies must apply the FACT to broader samples and populations other than university students in order to properly investigate and corroborate the proposed factorial structure.

Regarding the participants’ performance in each factor, four of the five statements that presented higher rates of “incorrect” or “I do not know” responses (statements 1, 3, 4, and 12) belong to the “fetal alcohol spectrum disorders” factor, while four of the five statements that presented the highest percentage of correct answers (statements 5, 7, 10, and 11) belong to the factor “conceptions and guidance on alcohol consumption during pregnancy.” These findings suggest that although students receive general information about the effects of alcohol during pregnancy, there is a lack of knowledge about fetal alcohol spectrum disorders.

In terms of external construct validity, the convergent analysis was conducted using the STD-KQ (47) because there was no other previously validated instrument on the same subject that could be utilized as a gold standard and allow concurrent validation. The positive and moderate correlation with the STD-KQ supports the validation of FACT and indicates that the greater the knowledge about sexually transmitted infections, the greater the knowledge about the effects of alcohol consumption during pregnancy.

The sample of university students showed higher knowledge on the subject when compared to other samples of non-pregnant women (26) and pregnant women (23, 73) from the general population. It can be hypothesized that the academic environment to which university students are exposed can help them acquire more knowledge on the subject. However, in relation to North American university students (29), the Brazilian sample seems to have a lower level of knowledge. When compared to professionals in the areas of health, education, and social services, Brazilian university students also showed less knowledge (74). These differences may have occurred because the samples were different, but also due to the lack of a unique, standardized instrument for assessment.

The greater knowledge among female participants coincides with the literature (32). The Brazilian sample also demonstrated results similar to those of other studies by pointing out that younger individuals (under 30 years of age) and older individuals (over 50 years of age) had less knowledge about the effects of alcohol use during pregnancy (22, 25). In relation to the number of living children and previous pregnancies, the present study differs from others by pointing out that women who have not had children or previous pregnancies have less knowledge on the subject when compared to those who have had living children and previous pregnancies (24, 25). There were statistically significant differences between FACT scores by type of high school, marital status, and field of study. Having attended a private school during high school, being married, and pursuing higher education in the area of health sciences were factors associated with a higher FACT score. Since the topics covered by FACT and those taught to students in this area are thematically related, it was already anticipated that students in the health field would score higher.

### 4.1 Limitations

This study has some limitations. The sample is of convenience and, therefore, does not necessarily represent the general population of Brazilian university students. There is also a bias concerning data collection, as only university students with internet access were able to respond to the questionnaire. There was an irregular distribution in the origin of the participants, with a predominance of respondents from the state of São Paulo and an absence of participants from three of the 27 Brazilian states (Amapá, Amazonas, and Rondônia). The absence of an analogous instrument, which can be considered a gold standard, prevented us from conducting concurrent validation, forcing us to carry out convergent validation with the STD-KQ, whose issue is different. Furthermore, FACT was developed and validated for the Brazilian sociocultural context, which restricts its application in other scenarios without prior cross-cultural adaptation.

## 5 Conclusion

This study developed and validated an easy-to-apply questionnaire to assess the knowledge of Brazilian university students about the effects of alcohol consumption during pregnancy. Based on the results of this study, the low level of knowledge among university students regarding alcohol consumption during pregnancy indicates the need for a better dialog between this population and healthcare professionals. In this regard, continuing healthcare education should be implemented, aiming to enhance the technical and communication skills of these professionals so that they can provide updated information in an accessible manner to young people. Implementing public health campaigns can also be a useful strategy for increasing the public’s knowledge of the potential harms associated with alcohol consumption on the fetus and, in turn, contributing to reducing population-level alcohol use during pregnancy.

The lack of awareness among the students and the general population regarding the consequences of alcohol consumption during pregnancy, the absence of an entirely safe level of alcohol consumption, and the misconceptions in the dissemination of knowledge on the subject make it an important area for further research. Therefore, we expect that new studies apply and validate the FACT in different sociocultural contexts to investigate more deeply the variables that can influence knowledge on the subject and identify new factors that may affect drinking behavior. Thus, the FACT can become a more robust tool and assist further investigation of this topic. In summary, using this tool, we expect to facilitate the development of more effective and culturally sensitive educational programs that may contribute to the primary prevention of fetal alcohol spectrum disorders and support the formulation and establishment of public policies in the area.

## Data availability statement

The original contributions presented in the study are included in the article/Supplementary material, further inquiries can be directed to the corresponding author.

## Ethics statement

The studies involving humans were approved by Human Research Ethics Committee at the Federal University of São Carlos (process CAAE 58094422.5.0000.5504). The studies were conducted in accordance with the local legislation and institutional requirements. The participants provided their written informed consent to participate in this study.

## Author contributions

GL: Conceptualization, Data curation, Formal analysis, Investigation, Methodology, Validation, Writing – original draft, Writing – review & editing. LA: Investigation, Validation, Writing – review & editing, Conceptualization. CG: Formal analysis, Investigation, Methodology, Validation, Writing – review & editing, Conceptualization, Supervision. DM: Conceptualization, Data curation, Formal analysis, Funding acquisition, Investigation, Methodology, Project administration, Supervision, Validation, Writing – original draft, Writing – review & editing.
