# Supplementary material for: Development and psychometric evaluation of a questionnaire to measure university students’ knowledge on the effects of alcohol use during pregnancy
Source: Front Public Health. 2024 May 10;12:1399333. doi: 10.3389/fpubh.2024.1399333 (PMC11116569; doi:10.3389/fpubh.2024.1399333)
Supplement: Supplementary file 1 [file Data_Sheet_1.DOCX]

Supplementary Material

**Development and psychometric evaluation of a questionnaire to measure university students’ knowledge on the effects of alcohol use during pregnancy**

**Guilherme Petek Ramos Leite^1^, Lucimar Retto da Silva de Avó^1,2^, Carla Maria Ramos Germano^1,2^, Débora Gusmão Melo^2,3*^**

^1^Departamento de Medicina, Universidade Federal de São Carlos (UFSCar), São Carlos, São Paulo, Brazil

^2^National Institute on Population Medical Genetics (INAGEMP), Porto Alegre, Rio Grande do Sul, Brazil

^2^Departamento de Morfologia e Genética, Escola Paulista de Medicina, Universidade Federal de São Paulo (UNIFESP), São Paulo, Brazil

***Correspondence:**Débora Gusmão Melo
[dgmelo@unifesp.br](mailto:dgmelo@unifesp.br)

**Appendix S1: Sociodemographic questionnaire**

Please answer the following inquiries about your sociodemographic profile.

1. How old are you? ______________

2. Where do you live in Brazil, city and state? ______________________

3. What gender are you? ( ) Female ( ) Male

4. Which country are you from?

( ) Brazil ( ) Other. Name: _____________________

5. What is your marital status?

( ) Married

( ) Common-law marriage

( ) Single

( ) Widowed

( ) Other. Describe: _____________________

6. What sexual orientation do you identify with?

( ) Heterosexual

( ) Homosexual

( ) Bisexual

( ) Pansexual

( ) Asexual

( ) Do not want to share the information

7. How many times have you given birth? (question only for females)

( ) 0

( ) one time

( ) two times

( ) three times or more

8. How many alive children do you have?

( ) 0

( ) one child

( ) two children

( ) three or more children

9. What kind of high school did you attend?

( ) Public

( ) Private

( ) Private and Public

10. What type of university/college do you currently attend?

( ) Private

( ) Public

11. Where does the course you're taking fit in?

( ) Math and Science

( ) Humanities

( ) Biological Sciences

( ) Health Sciences

12. How do you generally declare your skin color/ethnicity?

( ) White

( ) Black

( ) Asian

( ) Mixed

( ) Indigenous

13. How much does your family make each month in minimum wage (MW) terms? (If you are unemployed, take into account your nuclear family's income.)

( ) 1-3 MW

( ) 4-6 MW

( ) 7-10 MW

( ) 11-13 MW

( ) 14-16 MW

( ) 16 MW or more

14. What is your religious affiliation?

( ) Evangelical

( ) Catholic

( ) African-based religions

( ) No religion

( ) Other Describe: __________________________

**Table S1.** The first 45 true statements elaborated for FACT.

| **Nº** | **Statements in Portuguese** | **Statements in English** |
| --- | --- | --- |
| 1 | O álcool prejudica o desenvolvimento do feto durante a gravidez. | Alcohol is harmful to fetal development during pregnancy. |
| 2 | O consumo de álcool na gestação pode ocasionar abortamento. | Alcohol consumption during pregnancy can lead to miscarriage. |
| 3 | Qualquer quantidade de álcool consumido na gravidez pode prejudicar o feto. | Any amount of alcohol consumed during pregnancy can be harmful to the fetus. |
| 4 | Quanto mais álcool é consumido durante a gestação, maiores são as chances do feto apresentar algum problema. | The more alcohol is consumed during pregnancy, the higher the chances of the fetus having birth defects. |
| 5 | Quanto maior o teor alcoólico de uma bebida, mais prejudicial ela é para o desenvolvimento fetal. | The higher the alcohol content in a beverage, the more harmful it is to fetal development. |
| 6 | O álcool é mais prejudicial para o feto quando é consumido de vez em quando em grandes quantidades (ou seja, uso pesado e episódico de álcool). | Alcohol is more harmful to the fetus when it is consumed sporadically in large quantities (that is, heavy episodic drinking). |
| 7 | Qualquer tipo de bebida alcoólica é prejudicial ao desenvolvimento fetal. | Any kind of alcoholic beverage is harmful to fetal development. |
| 8 | Bebidas alcoólicas fermentadas (como cerveja e vinho) podem gerar danos ao feto quando consumidas na gestação. | Fermented drinks such as beer and wine can cause harm to the fetus when consumed during pregnancy. |
| 9 | Qualquer tipo de bebida alcoólica consumida durante a gestação pode gerar danos ao feto. | Any type of alcoholic beverage consumed during pregnancy can cause harm to the fetus. |
| 10 | O consumo de álcool é mais prejudicial para o feto durante o primeiro trimestre de gestação. | Alcohol consumption is the most harmful to the fetus during the first trimester of pregnancy. |
| 11 | O consumo de álcool na gestação pode ocasionar transtornos comportamentais na criança. | Alcohol consumption during pregnancy can lead to behavioral disorders in the child. |
| 12 | O consumo de álcool durante a gestação pode ocasionar transtornos emocionais na criança. | Alcohol consumption during pregnancy can lead to emotional disorders in the child. |
| 13 | O consumo de álcool durante a gestação pode ocasionar transtornos emocionais futuros no/a filho/filha na vida adulta. | Alcohol consumption during pregnancy can lead to future emotional disorders in the child's adult life. |
| 14 | O consumo de álcool durante a gestação pode ocasionar TDAH (Transtorno de Déficit de Atenção e Hiperatividade) na criança. | Alcohol consumption during pregnancy can lead to Attention Deficit Hyperactivity Disorder (ADHD) in the child. |
| 15 | Crianças cujas mães consumiram álcool na gravidez têm maior chance de terem o QI reduzido. | Children whose mothers consumed alcohol during pregnancy are more likely to have reduced IQ. |
| 16 | O álcool atravessa livremente a barreira placentária, entrando em contato direto com o feto. | Alcohol freely crosses the placental barrier, coming into direct contact with the fetus. |
| 17 | O álcool consumido durante a gestação pode diminuir a quantidade de nutrientes e vitaminas absorvidos pelo feto. | Alcohol consumption during pregnancy can reduce nutrients and vitamins absorption by the fetus. |
| 18 | O álcool consumido durante a gestação pode causar anemia no recém-nascido. | Alcohol consumption during pregnancy can cause anemia in a newborn. |
| 19 | O consumo de álcool na gravidez pode alterar o desenvolvimento, a função e a estrutura da placenta. | Alcohol consumption during pregnancy can alter the development, function, and structure of the placenta. |
| 20 | O álcool consumido durante a gestação pode causar baixo peso no feto. | Alcohol consumption during pregnancy can lead to a fetus with low birth weight. |
| 21 | O consumo de álcool na gestação pode causar malformação no cérebro do feto. | Alcohol consumption during pregnancy can cause congenital brain malformations. |
| 22 | O consumo de álcool na gestação pode causar microcefalia na criança. | Alcohol consumption during pregnancy can cause microcephaly in the child. |
| 23 | O consumo de álcool durante a gestação pode causar malformações faciais no feto. | Alcohol consumption during pregnancy can cause facial malformations in the fetus. |
| 24 | O consumo de álcool durante a gestação se relaciona com defeitos cardíacos congênitos na criança. | Alcohol consumption during pregnancy is related to congenital heart defects in the child. |
| 25 | O consumo de álcool durante a gestação se relaciona com malformações congênitas nos olhos da criança. | Alcohol consumption during pregnancy is related to congenital eye malformations in the child. |
| 26 | O consumo de álcool durante a gestação se relaciona com malformações renais e genitais na criança. | Alcohol consumption during pregnancy is related to kidney and genital malformations in the child. |
| 27 | O consumo de álcool durante a gestação se relaciona com malformações na coluna vertebral da criança. | Alcohol consumption during pregnancy is related to spinal column malformations in the child. |
| 28 | O consumo de álcool durante a gestação aumenta as chances da criança nascer com estrabismo. | Alcohol consumption during pregnancy increases the chances of the child being born with strabismus. |
| 29 | O consumo de álcool durante a gestação pode gerar malformações em diversas estruturas cerebrais da criança. | Alcohol consumption during pregnancy can cause malformations in several brain structures of the child. |
| 30 | Crianças cujas mães consumiram álcool na gravidez têm chance de serem mais baixas do que as crianças de mesma idade e sexo. | Children whose mothers consumed alcohol during pregnancy are expected to be shorter than children of the same age and sex. |
| 31 | O consumo de álcool durante a gestação pode causar um conjunto de condições irreversíveis na criança, incluindo defeitos congênitos e deficiência intelectual, que caracterizam a síndrome alcoólica fetal. | Alcohol consumption during pregnancy can cause a set of irreversible conditions in the child, including congenital defects and intellectual disability, which characterize Fetal Alcohol Syndrome. |
| 32 | A Síndrome Alcoólica Fetal é uma dentre várias consequências possíveis do consumo de álcool durante a gestação. | Fetal Alcohol Syndrome is one of several possible consequences of alcohol consumption during pregnancy. |
| 33 | O perfil genético da gestante e do feto pode interferir nos efeitos do álcool consumido durante a gestação. | The genetic profile of the pregnant woman and the fetus can interfere with the effects of alcohol consumed during pregnancy. |
| 34 | O Ministério da Saúde recomenda abstinência do uso de álcool durante a gestação. | The Ministry of Health recommends abstinence from alcohol use during pregnancy. |
| 35 | O Ministério da Saúde recomenda abstenção de bebida alcoólica no primeiro trimestre de gestação. | The Ministry of Health recommends abstaining from alcoholic beverages in the first trimester of pregnancy. |
| 36 | A literatura científica recomenda total abstinência de álcool durante toda a gestação. | Scientific literature recommends total abstinence from alcohol throughout pregnancy. |
| 37 | O consumo de álcool deve ser interrompido antes da gravidez, enquanto a mulher está tentando engravidar. | Alcohol consumption should be discontinued before pregnancy, while the woman is trying to conceive. |
| 38 | Caso o álcool seja consumido juntamente com o tabaco durante a gestação, as chances de dano no feto são aumentadas. | If alcohol is consumed along with tobacco during pregnancy, the chances of fetal damage are increased. |
| 39 | O álcool pode prejudicar o desenvolvimento do feto durante todo o período da gravidez. | Alcohol can harm fetal development throughout the entire pregnancy. |
| 40 | Qualquer quantidade de álcool pode prejudicar o desenvolvimento do feto durante todo o período da gravidez. | Any amount of alcohol can harm fetal development throughout the entire pregnancy. |
| 41 | Nenhuma bebida alcoólica é recomendada para aumentar a produção de leite materno. | No alcoholic beverage is recommended to increase breast milk production. |
| 42 | O consumo de álcool durante a gestação é prejudicial ao feto em todos os aspectos. | Alcohol consumption during pregnancy is harmful to the fetus in all aspects. |
| 43 | O consumo moderado de álcool não tem nenhum efeito benéfico para o feto. | Moderate alcohol consumption has no beneficial effects on the fetus. |
| 44 | O dano causado ao feto pelo uso de álcool pode ocorrer mesmo se os filhos de gestações anteriores não apresentarem problemas decorrentes do uso materno de álcool na gravidez. | Damage to the fetus from alcohol use can occur even if children from previous pregnancies did not have problems due to maternal alcohol consumption during pregnancy. |
| 45 | Ambos, o cigarro e o álcool, são prejudiciais ao feto. | Both cigarette smoking and alcohol are harmful to the fetus. |

**Table S2**. List of epistemological topics related to drinking while pregnant and how they align with the FACT questionnaire's statements.

| **Epistemological topics** | **Subthemes** | **Associated statements** |
| --- | --- | --- |
| Fetal hazards associated with alcohol usage during pregnancy | General risks of alcohol use during pregnancy | 1, 2 |
|  | Even though there is a dosage-effect link, there is no safe dose | 3, 4, 5, 6 |
|  | Alcoholic beverages of any kind are dangerous | 7, 8, 9 |
|  | Any gestational age is susceptible, with the highest risk occurring in the first trimester | 10 |
| Fetal alcohol spectrum disorders | Behavioral and neurodevelopmental disorders | 11, 12, 13, 14, 15 |
|  | Physiological issues with the maternal-fetal binomial system | 16, 17, 18, 19, 20 |
|  | Birth defects | 21, 22, 23, 24, 25, 26, 27, 28, 29, 30 |
|  | Fetal alcohol syndrome | 31, 32 |
|  | Genotype-phenotype interaction | 33 |
| Recommendations on drinking alcohol while pregnant | Guidelines from the Ministry of Health | 34, 35 |
|  | Complete abstinence when pregnant | 36 |
|  | Preconception health education on the topic (advice prior to conception) | 37, 38 |

**Table S3**. List of pregnancy-related drinking myths and misconceptions and their relation to the FACT questionnaire's statements.

| **Myths/Misconceptions** | **Associated statements** |
| --- | --- |
| It's okay to have a tiny amount | 39, 40 |
| Beer helps with breastfeeding | 41 |
| Alcohol has a relaxing effect on the fetus | 42 |
| The baby's immunity is boosted by alcohol since it "kills" infectious organisms in him/her | 43 |
| Behavior based on empirical evidence – drank in previous pregnancies and there was no negative outcome, so there are no problems | 44 |
| Smoking cigarettes may be more harmful than drinking alcohol | 45 |

**Table S4**. Values obtained from the initial 45 statements during the content validation stage with expert judges and their respective content validity index.

| **Statements** | | **Experts judges** | | | | | | | | | | | | | | | | | | | **CVI** |
| --- | --- | --- | --- | --- | --- | --- | --- | --- | --- | --- | --- | --- | --- | --- | --- | --- | --- | --- | --- | --- | --- |
|  |  | **1** | **2** | **3** | **4** | **5** | **6** | **7** | **8** | **9** | **10** | **11** | **12** | **13** | **14** | **15** | **16** | **17** | **18** | **19** |  |
| **1** | CL | 5 | 5 | 4 | 5 | 5 | 5 | 4 | 5 | 3 | 3 | 5 | 5 | 5 | 5 | 4 | 3 | 4 | 4 | 4 | 0.874 |
|  | PR | 5 | 5 | 5 | 5 | 5 | 5 | 5 | 5 | 5 | 5 | 5 | 5 | 5 | 5 | 5 | 5 | 5 | 5 | 5 | 1.000 |
| **2** | CL | 5 | 5 | 4 | 5 | 5 | 4 | 4 | 5 | 5 | 4 | 5 | 5 | 5 | 5 | 4 | 3 | 5 | 4 | 4 | 0.905 |
|  | PR | 5 | 5 | 5 | 5 | 5 | 5 | 5 | 5 | 5 | 4 | 5 | 4 | 5 | 5 | 5 | 5 | 5 | 5 | 5 | 0.979 |
| **3** | CL | 5 | 5 | 4 | 5 | 5 | 5 | 4 | 5 | 4 | 4 | 5 | 5 | 5 | 5 | 4 | 4 | 5 | 4 | 4 | 0.916 |
|  | PR | 5 | 5 | 5 | 5 | 5 | 5 | 5 | 5 | 4 | 5 | 5 | 5 | 5 | 5 | 5 | 5 | 5 | 4 | 5 | 0.979 |
| **4** | CL | 5 | 4 | 4 | 5 | 4 | 5 | 4 | 5 | 5 | 5 | 5 | 5 | 5 | 4 | 4 | 3 | 5 | 5 | 4 | 0.905 |
|  | PR | 5 | 5 | 5 | 5 | 5 | 5 | 5 | 5 | 5 | 5 | 5 | 4 | 5 | 4 | 5 | 5 | 5 | 5 | 5 | 0.979 |
| **5** | CL | 5 | 3 | 4 | 5 | 5 | 4 | 4 | 5 | 3 | 4 | 5 | 4 | 5 | 4 | 3 | 4 | 5 | 5 | 4 | 0.853 |
|  | PR | 5 | 5 | 4 | 5 | 5 | 3 | 5 | 5 | 5 | 5 | 5 | 4 | 5 | 4 | 4 | 5 | 5 | 5 | 5 | 0.937 |
| **6** | CL | 5 | 4 | 4 | 5 | 3 | 5 | 3 | 4 | 5 | 4 | 3 | 5 | 4 | 4 | 3 | 5 | 3 | 3 | 4 | 0.800 |
|  | PR | 5 | 5 | 5 | 5 | 4 | 5 | 5 | 4 | 4 | 5 | 5 | 4 | 4 | 4 | 5 | 5 | 4 | 5 | 5 | 0.926 |
| **7** | CL | 5 | 5 | 4 | 5 | 5 | 5 | 4 | 5 | 4 | 5 | 5 | 5 | 5 | 5 | 5 | 5 | 5 | 4 | 4 | 0.947 |
|  | PR | 5 | 5 | 5 | 5 | 5 | 5 | 5 | 5 | 5 | 5 | 5 | 5 | 5 | 5 | 5 | 5 | 5 | 5 | 5 | 1.000 |
| **8** | CL | 3 | 5 | 4 | 5 | 5 | 5 | 4 | 5 | 4 | 5 | 5 | 5 | 5 | 5 | 4 | 5 | 4 | 4 | 4 | 0.905 |
|  | PR | 3 | 5 | 5 | 5 | 3 | 5 | 4 | 5 | 5 | 5 | 5 | 5 | 4 | 5 | 5 | 5 | 4 | 5 | 4 | 0.916 |
| **9** | CL | 5 | 5 | 4 | 5 | 5 | 5 | 5 | 5 | 5 | 5 | 5 | 5 | 5 | 5 | 5 | 5 | 5 | 5 | 4 | 0.979 |
|  | PR | 5 | 5 | 5 | 5 | 5 | 5 | 5 | 5 | 5 | 5 | 5 | 5 | 5 | 5 | 5 | 5 | 5 | 5 | 4 | 0.989 |
| **10** | CL | 4 | 3 | 4 | 5 | 5 | 5 | 5 | 5 | 4 | 5 | 4 | 3 | 5 | 3 | 4 | 4 | 3 | 3 | 4 | 0.821 |
|  | PR | 5 | 5 | 5 | 5 | 5 | 5 | 5 | 5 | 5 | 5 | 5 | 4 | 4 | 5 | 4 | 5 | 3 | 3 | 4 | 0.916 |
| **11** | CL | 4 | 4 | 4 | 2 | 5 | 1 | 3 | 5 | 4 | 4 | 5 | 5 | 4 | 4 | 5 | 5 | 4 | 5 | 5 | 0.821 |
|  | PR | 5 | 5 | 5 | 5 | 5 | 5 | 5 | 5 | 5 | 5 | 5 | 5 | 5 | 5 | 5 | 5 | 5 | 5 | 5 | 1.000 |
| **12** | CL | 3 | 4 | 4 | 2 | 5 | 5 | 3 | 5 | 4 | 5 | 5 | 3 | 4 | 4 | 3 | 5 | 5 | 5 | 5 | 0.832 |
|  | PR | 5 | 5 | 5 | 5 | 5 | 5 | 5 | 5 | 4 | 5 | 5 | 4 | 4 | 5 | 3 | 5 | 5 | 5 | 5 | 0.947 |
| **13** | CL | 5 | 4 | 5 | 2 | 5 | 5 | 3 | 5 | 4 | 5 | 4 | 4 | 4 | 4 | 2 | 5 | 5 | 5 | 5 | 0.853 |
|  | PR | 5 | 5 | 4 | 5 | 4 | 5 | 5 | 5 | 2 | 5 | 5 | 5 | 4 | 5 | 2 | 5 | 5 | 5 | 5 | 0.905 |
| **14** | CL | 5 | 5 | 4 | 2 | 5 | 5 | 5 | 5 | 4 | 5 | 5 | 5 | 5 | 5 | 5 | 5 | 5 | 5 | 5 | 0.947 |
|  | PR | 5 | 5 | 5 | 5 | 5 | 5 | 5 | 5 | 4 | 5 | 5 | 5 | 4 | 5 | 5 | 5 | 5 | 5 | 4 | 0.968 |
| **15** | CL | 3 | 3 | 4 | 2 | 4 | 5 | 4 | 5 | 3 | 5 | 4 | 5 | 5 | 3 | 2 | 4 | 4 | 5 | 4 | 0.779 |
|  | PR | 5 | 5 | 5 | 5 | 5 | 5 | 5 | 5 | 2 | 5 | 5 | 5 | 4 | 5 | 5 | 5 | 5 | 5 | 5 | 0.958 |
| **16** | CL | 4 | 2 | 2 | 5 | 5 | 5 | 5 | 5 | 4 | 3 | 5 | 4 | 4 | 3 | 4 | 3 | 3 | 5 | 4 | 0.789 |
|  | PR | 5 | 5 | 5 | 5 | 5 | 5 | 5 | 5 | 5 | 5 | 5 | 5 | 4 | 5 | 5 | 5 | 5 | 5 | 5 | 0.989 |
| **17** | CL | 5 | 4 | 4 | 5 | 5 | 5 | 5 | 5 | 4 | 5 | 5 | 4 | 5 | 5 | 4 | 5 | 4 | 5 | 4 | 0.926 |
|  | PR | 5 | 5 | 5 | 5 | 5 | 5 | 5 | 5 | 5 | 5 | 5 | 5 | 5 | 4 | 5 | 5 | 5 | 5 | 4 | 0.979 |
| **18** | CL | 5 | 4 | 4 | 5 | 5 | 5 | 5 | 5 | 5 | 5 | 5 | 5 | 5 | 5 | 5 | 5 | 5 | 4 | 5 | 0.968 |
|  | PR | 5 | 3 | 4 | 5 | 5 | 5 | 5 | 5 | 5 | 4 | 5 | 5 | 3 | 4 | 4 | 5 | 5 | 3 | 4 | 0.884 |
| **19** | CL | 4 | 3 | 4 | 5 | 5 | 5 | 4 | 5 | 3 | 5 | 5 | 4 | 5 | 4 | 4 | 4 | 4 | 4 | 4 | 0.853 |
|  | PR | 5 | 3 | 2 | 5 | 5 | 5 | 3 | 5 | 5 | 5 | 5 | 4 | 3 | 4 | 4 | 5 | 5 | 5 | 4 | 0.863 |
| **20** | CL | 5 | 4 | 4 | 2 | 4 | 5 | 5 | 5 | 4 | 5 | 5 | 5 | 5 | 5 | 5 | 5 | 5 | 5 | 4 | 0.916 |
|  | PR | 5 | 5 | 4 | 5 | 4 | 5 | 5 | 5 | 5 | 5 | 5 | 5 | 5 | 5 | 5 | 5 | 5 | 5 | 4 | 0.968 |
| **21** | CL | 5 | 5 | 4 | 5 | 5 | 5 | 4 | 5 | 4 | 5 | 5 | 5 | 5 | 5 | 5 | 5 | 5 | 5 | 4 | 0.958 |
|  | PR | 5 | 5 | 5 | 5 | 5 | 5 | 4 | 5 | 5 | 5 | 5 | 5 | 5 | 5 | 5 | 5 | 5 | 5 | 5 | 0.989 |
| **22** | CL | 4 | 4 | 4 | 3 | 5 | 3 | 5 | 5 | 3 | 5 | 5 | 5 | 5 | 4 | 5 | 5 | 5 | 5 | 5 | 0.895 |
|  | PR | 5 | 5 | 5 | 5 | 4 | 5 | 5 | 5 | 5 | 5 | 5 | 5 | 5 | 4 | 5 | 5 | 5 | 5 | 4 | 0.968 |
| **23** | CL | 5 | 3 | 4 | 5 | 5 | 3 | 4 | 5 | 4 | 3 | 5 | 5 | 5 | 4 | 5 | 4 | 3 | 4 | 4 | 0.842 |
|  | PR | 5 | 3 | 5 | 5 | 4 | 5 | 5 | 5 | 4 | 5 | 5 | 5 | 5 | 3 | 5 | 5 | 4 | 4 | 4 | 0.905 |
| **24** | CL | 5 | 3 | 4 | 5 | 5 | 5 | 5 | 5 | 3 | 4 | 5 | 4 | 5 | 5 | 3 | 5 | 4 | 5 | 5 | 0.895 |
|  | PR | 5 | 5 | 5 | 5 | 5 | 5 | 5 | 5 | 4 | 5 | 5 | 5 | 4 | 5 | 5 | 5 | 5 | 4 | 4 | 0.958 |
| **25** | CL | 5 | 3 | 4 | 5 | 4 | 3 | 4 | 5 | 5 | 4 | 5 | 5 | 5 | 5 | 3 | 5 | 3 | 4 | 5 | 0.863 |
|  | PR | 5 | 5 | 5 | 5 | 4 | 5 | 5 | 5 | 4 | 4 | 5 | 5 | 4 | 5 | 4 | 5 | 4 | 4 | 4 | 0.916 |
| **26** | CL | 5 | 3 | 4 | 5 | 5 | 3 | 5 | 5 | 3 | 4 | 5 | 5 | 5 | 5 | 4 | 5 | 4 | 4 | 5 | 0.884 |
|  | PR | 5 | 5 | 5 | 5 | 4 | 5 | 5 | 5 | 3 | 5 | 5 | 5 | 4 | 5 | 4 | 5 | 5 | 3 | 4 | 0.916 |
| **27** | CL | 5 | 4 | 4 | 5 | 5 | 5 | 5 | 5 | 4 | 5 | 5 | 5 | 5 | 5 | 3 | 5 | 3 | 4 | 5 | 0.916 |
|  | PR | 5 | 5 | 5 | 5 | 4 | 5 | 5 | 5 | 5 | 5 | 5 | 5 | 4 | 5 | 4 | 5 | 4 | 2 | 4 | 0.916 |
| **28** | CL | 4 | 5 | 4 | 3 | 5 | 5 | 5 | 5 | 4 | 5 | 5 | 5 | 5 | 5 | 5 | 5 | 5 | 4 | 5 | 0.937 |
|  | PR | 5 | 5 | 3 | 5 | 4 | 5 | 4 | 5 | 2 | 5 | 5 | 3 | 3 | 4 | 4 | 5 | 5 | 4 | 4 | 0.842 |
| **29** | CL | 5 | 4 | 4 | 5 | 5 | 5 | 4 | 5 | 5 | 5 | 5 | 5 | 5 | 5 | 4 | 5 | 4 | 5 | 5 | 0.947 |
|  | PR | 5 | 5 | 5 | 5 | 4 | 5 | 5 | 5 | 5 | 5 | 5 | 5 | 3 | 5 | 5 | 5 | 5 | 5 | 4 | 0.958 |
| **30** | CL | 5 | 4 | 4 | 5 | 4 | 5 | 5 | 5 | 5 | 5 | 3 | 5 | 5 | 3 | 5 | 5 | 5 | 4 | 5 | 0.916 |
|  | PR | 5 | 5 | 5 | 5 | 4 | 5 | 5 | 5 | 3 | 5 | 5 | 3 | 3 | 4 | 5 | 5 | 5 | 4 | 4 | 0.895 |
| **31** | CL | 5 | 4 | 4 | 5 | 4 | 5 | 5 | 5 | 5 | 4 | 5 | 5 | 5 | 5 | 5 | 5 | 4 | 5 | 5 | 0.947 |
|  | PR | 5 | 5 | 4 | 5 | 5 | 5 | 5 | 5 | 5 | 5 | 5 | 5 | 5 | 5 | 5 | 5 | 5 | 5 | 5 | 0.989 |
| **32** | CL | 5 | 4 | 5 | 5 | 4 | 5 | 5 | 5 | 3 | 5 | 5 | 5 | 5 | 5 | 5 | 5 | 3 | 4 | 5 | 0.926 |
|  | PR | 5 | 5 | 5 | 5 | 5 | 5 | 5 | 5 | 5 | 5 | 5 | 5 | 4 | 5 | 5 | 5 | 5 | 5 | 4 | 0.979 |
| **33** | CL | 5 | 3 | 4 | 2 | 4 | 5 | 4 | 5 | 2 | 3 | 4 | 5 | 5 | 4 | 4 | 4 | 3 | 4 | 4 | 0.779 |
|  | PR | 5 | 3 | 4 | 5 | 5 | 5 | 5 | 5 | 3 | 5 | 5 | 4 | 4 | 5 | 5 | 4 | 3 | 5 | 3 | 0.874 |
| **34** | CL | 4 | 3 | 4 | 5 | 4 | 5 | 5 | 5 | 5 | 5 | 5 | 5 | 5 | 4 | 4 | 5 | 3 | 5 | 5 | 0.905 |
|  | PR | 5 | 5 | 5 | 5 | 5 | 5 | 5 | 5 | 5 | 5 | 5 | 5 | 5 | 5 | 5 | 5 | 5 | 5 | 4 | 0.989 |
| **35** | CL | 4 | 3 | 5 | 5 | 3 | 5 | 5 | 5 | 4 | 5 | 3 | 4 | 5 | 4 | 3 | 4 | 4 | 3 | 5 | 0.832 |
|  | PR | 5 | 5 | 5 | 5 | 3 | 4 | 4 | 5 | 5 | 5 | 5 | 3 | 4 | 4 | 4 | 5 | 3 | 3 | 4 | 0.853 |
| **36** | CL | 5 | 4 | 4 | 5 | 5 | 5 | 5 | 5 | 5 | 3 | 5 | 5 | 5 | 4 | 4 | 5 | 4 | 4 | 5 | 0.916 |
|  | PR | 5 | 5 | 5 | 5 | 5 | 5 | 5 | 5 | 5 | 5 | 5 | 5 | 4 | 5 | 5 | 5 | 5 | 4 | 4 | 0.968 |
| **37** | CL | 3 | 3 | 4 | 5 | 5 | 5 | 5 | 5 | 4 | 5 | 3 | 5 | 5 | 4 | 5 | 5 | 5 | 4 | 5 | 0.895 |
|  | PR | 5 | 5 | 5 | 5 | 5 | 5 | 5 | 5 | 4 | 5 | 5 | 5 | 5 | 5 | 5 | 5 | 5 | 5 | 4 | 0.979 |
| **38** | CL | 5 | 4 | 4 | 5 | 4 | 5 | 5 | 5 | 5 | 5 | 5 | 5 | 5 | 4 | 5 | 4 | 5 | 4 | 5 | 0.937 |
|  | PR | 5 | 5 | 5 | 5 | 4 | 5 | 5 | 5 | 4 | 3 | 5 | 5 | 5 | 5 | 5 | 5 | 5 | 5 | 4 | 0.947 |
| **39** | CL | 5 | 4 | 4 | 5 | 5 | 5 | 5 | 5 | 5 | 5 | 5 | 5 | 5 | 5 | 3 | 5 | 5 | 5 | 5 | 0.958 |
|  | PR | 5 | 5 | 5 | 5 | 5 | 5 | 5 | 5 | 5 | 5 | 5 | 5 | 5 | 5 | 5 | 5 | 5 | 5 | 4 | 0.989 |
| **40** | CL | 5 | 5 | 4 | 5 | 5 | 5 | 5 | 5 | 4 | 3 | 5 | 5 | 5 | 5 | 5 | 5 | 5 | 4 | 4 | 0.937 |
|  | PR | 5 | 5 | 5 | 5 | 5 | 5 | 5 | 5 | 4 | 5 | 5 | 5 | 5 | 5 | 5 | 5 | 5 | 5 | 4 | 0.979 |
| **41** | CL | 5 | 4 | 5 | 5 | 4 | 5 | 5 | 5 | 5 | 5 | 5 | 5 | 5 | 5 | 5 | 5 | 5 | 5 | 5 | 0.979 |
|  | PR | 5 | 5 | 5 | 5 | 5 | 5 | 5 | 5 | 5 | 5 | 5 | 5 | 4 | 5 | 5 | 5 | 5 | 5 | 4 | 0.979 |
| **42** | CL | 4 | 5 | 4 | 5 | 5 | 5 | 5 | 5 | 2 | 5 | 3 | 4 | 3 | 5 | 5 | 5 | 5 | 4 | 4 | 0.874 |
|  | PR | 5 | 5 | 5 | 5 | 4 | 5 | 5 | 5 | 2 | 5 | 5 | 4 | 3 | 5 | 5 | 5 | 5 | 5 | 4 | 0.916 |
| **43** | CL | 5 | 3 | 2 | 3 | 3 | 5 | 3 | 5 | 4 | 5 | 5 | 5 | 4 | 5 | 2 | 5 | 2 | 4 | 4 | 0.779 |
|  | PR | 5 | 5 | 1 | 5 | 4 | 5 | 5 | 5 | 5 | 5 | 5 | 5 | 3 | 5 | 2 | 5 | 2 | 5 | 4 | 0.853 |
| **44** | CL | 5 | 4 | 5 | 5 | 3 | 5 | 5 | 5 | 4 | 5 | 5 | 5 | 5 | 3 | 4 | 5 | 4 | 4 | 4 | 0.895 |
|  | PR | 5 | 5 | 5 | 5 | 5 | 5 | 5 | 5 | 5 | 5 | 5 | 5 | 4 | 5 | 5 | 5 | 3 | 5 | 4 | 0.958 |
| **45** | CL | 4 | 5 | 4 | 5 | 5 | 5 | 5 | 5 | 5 | 5 | 5 | 5 | 4 | 4 | 3 | 5 | 5 | 5 | 4 | 0.926 |
|  | PR | 5 | 5 | 5 | 5 | 5 | 5 | 5 | 5 | 5 | 5 | 5 | 5 | 4 | 5 | 4 | 5 | 5 | 5 | 4 | 0.968 |

*CL = clarity of language / PR = practical relevance

**Table S5.** CVI scores for each of the 45 statements in the original FACT in terms of language clarity.

| **Ranking** | **CVI scores** | **Statements (1 to 45)** |
| --- | --- | --- |
| 1st | 0.979 | 9, 41 |
| 2nd | 0.968 | 18 |
| 3rd | 0.958 | 21, 39 |
| 4th | 0.947 | 7, 14, 29, 31 |
| 5th | 0.937 | 28, 38, 40 |
| 6th | 0.926 | 17, 32, 45 |
| 7th | 0.916 | 3, 20, 27, 30, 36 |
| 8th | 0.905 | 2, 4, 8, 34 |
| 9th | 0.895 | 22, 24, 37, 44 |
| 10th | 0.884 | 26 |
| 11th | 0.874 | 1, 42 |
| 12th | 0.863 | 25 |
| 13th | 0.853 | 5, 13, 19 |
| 14th | 0.842 | 23 |
| 15th | 0.832 | 12, 35 |
| 16th | 0.821 | 10, 11 |
| 17th | 0.800 | 6 |
| 18th | 0.789 | 16 |
| 19th | 0.779 | 15, 33, 43 |

**Table S6**. CVI scores for each of the 45 statements in the original FACT in terms of practical relevance.

| **Ranking** | **CVI scores** | **Statements (1 to 45)** |
| --- | --- | --- |
| 1st | 1.000 | 1, 7, 11 |
| 2nd | 0.989 | 9, 16, 21, 31, 34, 39 |
| 3rd | 0.979 | 2, 3, 4, 17, 32, 37, 40, 41 |
| 4th | 0.968 | 14, 20, 22, 36, 45 |
| 5th | 0.958 | 15, 24, 29, 44 |
| 6th | 0.947 | 12, 38 |
| 7th | 0.937 | 5 |
| 8th | 0.926 | 6 |
| 9th | 0.916 | 8, 10, 25, 26, 27, 42 |
| 10th | 0.905 | 13, 23 |
| 11th | 0.895 | 30 |
| 12th | 0.884 | 18 |
| 13th | 0.874 | 33 |
| 14th | 0.863 | 19 |
| 15th | 0.853 | 35, 43 |
| 16th | 0.842 | 28 |

**Table S7**. The percentage of theoretical adequacy of the 45 statements in FACT's first edition.

| **Ranking** | **Theoretical adequacy** **(in %)** | **Statements (1 to 45)** |
| --- | --- | --- |
| 1st | 100 | 1, 2, 3, 7, 9, 11, 14, 16, 17, 19, 22, 30, 31, 32, 33, 34, 36, 39, 40, 41, 42, 44, 45 |
| 2nd | 95 | 4, 8, 15, 18, 20, 24, 25, 37, 38 |
| 3rd | 89 | 5, 12, 21, 23, 28, 29, 43 |
| 4th | 84 | 10, 13 |
| 5th | 79 | 26, 27 |
| 6th | 58 | 6 |
| 7th | 53 | 35 |

**Table S8**. FACT after expert content validation. The letter "F" stands for false statements, whereas the letter "T" stands for true statements.

| **No** | **Statements in Portuguese** | **Statements in English** |
| --- | --- | --- |
| 1 | O álcool é uma substância que não faz mal para o bebê e pode ser consumido na gravidez (F). | Alcohol is a substance that does not harm the baby and can be consumed during pregnancy (F). |
| 2 | Existem alguns tipos de bebidas alcoólicas que não fazem mal para o bebê e podem ser consumidas durante a gravidez (F). | There are some types of alcoholic beverages that do not harm the baby and can be consumed during pregnancy (F). |
| 3 | Tomar bebida alcoólica durante a gravidez pode causar problemas permanentes na criança, incluindo defeitos físicos e deficiência intelectual, que caracterizam a síndrome alcoólica fetal (V). | Consuming alcohol during pregnancy can cause permanent problems in the child, including physical disorders and intellectual disability, which characterize fetal alcohol syndrome (T). |
| 4 | Mães que beberam na gravidez podem ter crianças com problemas no desenvolvimento neurológico, como falta de atenção e hiperatividade (V). | Mothers who had alcohol during pregnancy may have children with neurological development disorders, such as attention deficit and hyperactivity (T). |
| 5 | Mães que beberam na gravidez podem ter crianças com problemas emocionais e de comportamento, como ataques de raiva ou choro, ansiedade e agressividade (V). | Mothers who had alcohol during pregnancy may have children with emotional and behavioral problems, such as tantrums, crying, anxiety, and aggressiveness (T). |
| 6 | Mães que beberam na gravidez podem ter crianças com inteligência abaixo do normal (V). | Mothers who had alcohol during pregnancy may have children with below-average intelligence (T). |
| 7 | Qualquer quantidade de álcool consumido pela gestante pode prejudicar o bebê, portanto, não há dose segura (V). | Any amount of alcohol consumed by a pregnant woman can harm the baby, so there is no safe amount of alcohol consumption (T). |
| 8 | A bebida alcoólica ingerida durante a gravidez não interfere na quantidade de alimentos e vitaminas absorvidos pelo bebê, não atrapalhando assim seu ganho de peso (F). | Alcoholic beverages consumed during pregnancy do not affect the amount of food and vitamins absorbed by the baby, thus not interfering with its weight gain (F). |
| 9 | Os estudos científicos recomendam não consumir nenhuma quantidade de álcool durante todo o período da gravidez (V). | Scientific studies recommend not consuming any amount of alcohol throughout the entire pregnancy (T). |
| 10 | Tomar bebida alcoólica durante a gravidez pode causar diminuição da cabeça na criança (microcefalia) (V). | Consuming alcoholic beverages during pregnancy can cause a reduction in the size of the baby's head (microcephaly) (T). |
| 11 | Tomar bebida alcoólica durante a gravidez pode causar defeitos no coração da criança (V). | Consuming alcoholic beverages during pregnancy can cause heart defects in the child (T). |
| 12 | Não há recomendações do Ministério da Saúde sobre uso de álcool na gravidez (F). | There are no recommendations from the Ministry of Health regarding alcohol use during pregnancy (F). |
| 13 | Mulheres que estão tentando engravidar devem parar de beber álcool (V). | Women trying to conceive should stop drinking alcohol (T). |
| 14 | O uso de bebidas alcoólicas por mulheres grávidas pode prejudicar o desenvolvimento do bebê apenas nos três primeiros meses de gravidez (F). | Pregnant women drinking alcoholic beverages can harm the baby's development only in the first three months of pregnancy (F). |
| 15 | Mulheres que amamentam não devem usar nenhum tipo de bebida alcoólica (V). | Women who are breastfeeding should not consume any type of alcoholic beverage (T). |
| 16 | Se uma mulher bebeu durante a sua gravidez e seu filho nasceu saudável, indica que ela pode continuar bebendo nas próximas gestações pois não existe chance dela ter um filho com problemas relacionados ao álcool (F). | If a woman had alcohol during her pregnancy, and her child was born healthy, it does not indicate that she can continue drinking in subsequent pregnancies, as there is still a risk of her having a child with alcohol-related problems (F). |
| 17 | Uso de bebida alcoólica junto com cigarro durante a gestação aumenta as chances de problemas no bebê (V). | Consuming alcoholic beverages along with smoking during pregnancy increases the chances of problems in the baby (T). |

**Table S9**. Average of level of understanding for the 17 FACT assertions.

| **Statements** | **Scores** | **Standard deviation** |
| --- | --- | --- |
| 1 | 4.00 | ± 0.00 |
| 2 | 3.84 | ± 0.52 |
| 3 | 3.94 | ± 0.25 |
| 4 | 3.87 | ± 0.34 |
| 5 | 3.84 | ± 0.58 |
| 6 | 3.87 | ± 0.43 |
| 7 | 4.00 | ± 0.00 |
| 8 | 3.90 | ± 0.30 |
| 9 | 3.90 | ± 0.40 |
| 10 | 3.94 | ± 0.25 |
| 11 | 4.00 | ± 0.00 |
| 12 | 3.84 | ± 0.52 |
| 13 | 3.97 | ± 0.18 |
| 14 | 3.94 | ± 0.25 |
| 15 | 4.00 | ± 0.00 |
| 16 | 3.77 | ± 0.80 |
| 17 | 3.97 | ± 0.18 |

**Table S10**. FACT's semi-final version, after an evaluation of level of understanding. The letter "F" stands for false statements, whereas the letter "T" stands for true statements.

| Nº | Statements in Portuguese | Statements in English |
| --- | --- | --- |
| 1 | O álcool é uma substância que não faz mal para o bebê e pode ser consumido na gravidez. (F) | Alcohol is a substance that does not harm the baby and can be consumed during pregnancy. (F) |
| 2 | Tomar bebida alcoólica durante a gravidez pode causar defeitos no coração da criança. (V) | Consuming alcoholic beverages during pregnancy can cause heart defects in the child. (T) |
| 3 | Tomar bebida alcoólica durante a gravidez pode causar problemas permanentes na criança, incluindo defeitos físicos e deficiência intelectual, que caracterizam a síndrome alcoólica fetal. (V) | Consuming alcohol during pregnancy can cause permanent problems in the child, including physical disorders and intellectual disability, which characterize fetal alcohol syndrome. (T) |
| 4 | Mães que beberam álcool na gravidez podem ter crianças com inteligência abaixo do normal. (V) | Mothers who had alcohol during pregnancy may have children with below-average intelligence. (T) |
| 5 | A bebida alcoólica ingerida durante a gravidez não interfere na quantidade de alimentos e vitaminas absorvidos pelo bebê, não atrapalhando seu ganho de peso. (F) | Alcoholic beverages consumed during pregnancy do not affect the amount of food and vitamins absorbed by the baby, thus not interfering with its weight gain. (F) |
| 6 | Mães que beberam álcool na gravidez podem ter crianças com problemas emocionais e de comportamento, como ataques de raiva ou choro, ansiedade e agressividade. (V) | Mothers who had alcohol during pregnancy may have children with emotional and behavioral problems, such as tantrums, crying, anxiety, and aggressiveness. (T) |
| 7 | Se uma mulher bebeu álcool durante a gravidez e seu filho nasceu saudável, isso indica que ela pode beber nas próximas gestações sem qualquer risco para a criança. (F) | If a woman had alcohol during her pregnancy, and her child was born healthy, it indicates that she can continue drinking it in subsequent pregnancies, as there are no risks for the child. (F) |
| 8 | Qualquer quantidade de álcool consumido pela gestante pode prejudicar o bebê, portanto, não há dose segura. (V) | Any amount of alcohol consumed by a pregnant woman can harm the baby, so there is no safe amount of alcohol consumption. (T) |
| 9 | Qualquer tipo de bebida alcoólica consumida durante a gravidez pode prejudicar o desenvolvimento do bebê. (V) | Any type of alcohol consumed during pregnancy can be harmful to the baby’s development. (T) |
| 10 | Mães que beberam álcool na gravidez podem ter crianças com problemas no desenvolvimento neurológico, como falta de atenção e hiperatividade. (V) | Mothers who had alcohol during pregnancy may have children with neurological development disorders, such as attention deficit and hyperactivity. (T) |
| 11 | A cerveja preta melhora a quantidade do leite materno. (F) | Dark beer improves breast milk production. (F) |
| 12 | Os estudos científicos recomendam não consumir qualquer quantidade de álcool durante todo o período da gravidez. (V) | Scientific studies recommend not consuming any amount of alcohol throughout the entire pregnancy. (T) |
| 13 | Não há recomendação oficial do Ministério da Saúde sobre uso de álcool na gravidez. (F) | There are no official recommendations from the Ministry of Health regarding alcohol use during pregnancy. (F) |
| 14 | O consumo de bebida alcoólica junto com cigarro durante a gestação aumenta a chance do bebê nascer com problemas. (V) | Consuming alcoholic beverages along with smoking during pregnancy increases the chances of problems in the baby. (T) |
| 15 | O consumo de bebidas alcoólicas por mulheres grávidas pode prejudicar o desenvolvimento do bebê apenas nos três primeiros meses de gravidez. (F) | Pregnant women’s consumption of alcoholic beverages can harm the baby's development only in the first three months of pregnancy. (F) |
| 16 | Tomar bebida alcoólica durante a gravidez pode causar diminuição da cabeça na criança (microcefalia). (V) | Consuming alcoholic beverages during pregnancy can cause a reduction in the size of the baby's head (microcephaly). (T) |
| 17 | Mulheres que estão tentando engravidar devem parar de beber álcool. (V) | Women trying to conceive should stop drinking alcohol. (T) |

**Table S11.** Sociodemographic information from participants (n = 768).

| **Sociodemographic variables** | **n** | **%** |
| --- | --- | --- |
| **Gender** | | |
| Female | 554 | 72.14% |
| Male | 214 | 27.86% |
| **Age** | | |
| 18 - 30 years | 675 | 87.89% |
| 31 – 40 years | 67 | 8.72% |
| 41 - 50 years | 17 | 2.21% |
| Over 51 years | 9 | 1.17% |
| **Skin color/ethnicity** | | |
| White | 515 | 67.06% |
| Black | 50 | 6.51% |
| Mixed-race | 166 | 21.62% |
| Asian | 31 | 4.04% |
| Indigenous | 6 | 0.78% |
| **Sexual orientation** | | |
| Heterosexual | 510 | 66.41% |
| Homosexual | 51 | 6.64% |
| Bisexual | 151 | 19.66% |
| Pansexual | 18 | 2.34% |
| Asexual | 19 | 2.47% |
| Do not want to share the information | 19 | 2.47% |
| **Marital status** | | |
| Single | 602 | 78.39% |
| Married | 72 | 9.38% |
| Common-law marriage | 86 | 11.20% |
| Divorced | 8 | 1.04% |
| **Previous pregnancies (females only)** |  |  |
| 0 | 484 | 87.36% |
| 1 | 45 | 8.12% |
| 2 | 17 | 3.07% |
| 3 or more | 8 | 1.44% |
| **Alive children** |  |  |
| 0 | 682 | 88.80% |
| 1 | 58 | 7.55% |
| 2 | 21 | 2.73% |
| 3 or more | 7 | 0.91% |
| **Type of high school** | | |
| Public | 345 | 44.92% |
| Private | 392 | 51.04% |
| Public and Private | 31 | 4.04% |
| **Type of university/college** | | |
| Public | 693 | 90.23% |
| Private | 75 | 9.77% |
| **Field of study** | | |
| Health Sciences | 253 | 32.94% |
| Math and Science | 221 | 28.78% |
| Humanities | 161 | 20.96% |
| Biological Sciences | 133 | 17.32% |
| **Monthly income (MW = minimum wage)** | | |
| 1-3 MW | 291 | 37.89% |
| 4-6 MW | 219 | 28.52% |
| 7-10 MW | 120 | 15.63% |
| 11-13 MW | 54 | 7.03% |
| 14-16 MW | 23 | 2.99% |
| Over 16 MW | 61 | 7.94% |
| **Religion** | | |
| Catholic | 221 | 28.78% |
| Evangelical | 108 | 14.06% |
| African-based religion | 25 | 3.26% |
| Non-religious | 349 | 45.44% |
| Others | 65 | 8.46% |
| **Region** | | |
| Southeast | 520 | 67.71% |
| South | 45 | 5.86% |
| Midwest | 62 | 8.07% |
| Northeast | 102 | 13.28% |
| North | 39 | 5.08% |
| **State of origin** | | |
| São Paulo | 496 | 64.58% |
| Rio de Janeiro | 2 | 0.26% |
| Minas Gerais | 20 | 2.60% |
| Espírito Santo | 2 | 0.26% |
| Rio Grande do Sul | 16 | 2.08% |
| Santa Catarina | 6 | 0.78% |
| Paraná | 23 | 3.00% |
| Distrito Federal | 34 | 4.43% |
| Mato Grosso | 2 | 0.26% |
| Mato Grosso do Sul | 20 | 2.60% |
| Goiás | 6 | 0.78% |
| Bahia | 6 | 0.78% |
| Sergipe | 1 | 0.13% |
| Alagoas | 33 | 4.30% |
| Pernambuco | 27 | 3.51% |
| Paraíba | 4 | 0.52% |
| Rio Grande do Norte | 11 | 1.43% |
| Ceará | 17 | 2.21% |
| Piauí | 2 | 0.26% |
| Maranhão | 1 | 0.13% |
| Tocantins | 7 | 0.91% |
| Pará | 1 | 0.13% |
| Amapá | 0 | 0.00% |
| Amazonas | 0 | 0.00% |
| Roraima | 9 | 1.17% |
| Rondônia | 0 | 0.00% |
| Acre | 22 | 2.87% |

**Table S12**. Cronbach's alpha index of the total FACT and its 12 items (n = 768).

| **FACT statements** | **Cronbach's alpha index** |
| --- | --- |
| 1 | 0.7613 |
| 2 | 0.7553 |
| 3 | 0.7520 |
| 4 | 0.7611 |
| 5 | 0.7827 |
| 6 | 0.7602 |
| 7 | 0.7537 |
| 8 | 0.7473 |
| 9 | 0.7632 |
| 10 | 0.7620 |
| 11 | 0.7615 |
| 12 | 0.7601 |
| **Total** | 0.7976 |

**Table S13**. Cronbach's alpha index of the total STD-KQ and its 23 items (n = 768).

| **STD-KQ statements** | **Cronbach's alpha index** |
| --- | --- |
| 1 | 0.8380 |
| 2 | 0.8435 |
| 3 | 0.8372 |
| 4 | 0.8416 |
| 5 | 0.8432 |
| 6 | 0.8454 |
| 7 | 0.8442 |
| 8 | 0.8397 |
| 9 | 0.8405 |
| 10 | 0.8415 |
| 11 | 0.8407 |
| 12 | 0.8462 |
| 13 | 0.8434 |
| 14 | 0.8412 |
| 15 | 0.8455 |
| 16 | 0.8489 |
| 17 | 0.8401 |
| 18 | 0.8411 |
| 19 | 0.8422 |
| 20 | 0.8381 |
| 21 | 0.8429 |
| 22 | 0.8441 |
| 23 | 0.8443 |
| **Total** | 0.8653 |
